# Synergistic Engineering of Pyrene–Thiazolothiazole-Based Donor−π–Acceptor Conjugated Microporous Polymers with Heteroatom Embedding for Efficient Visible-Light Photocatalyst for Organic Dye Degradation

**DOI:** 10.1021/acspolymersau.5c00083

**Published:** 2025-09-11

**Authors:** Yang-Chin Kao, Mohamed Gamal Mohamed, Ying-Hong Chen, Mohsin Ejaz, Shiao-Wei Kuo

**Affiliations:** † Department of Materials and Optoelectronic Science, Center for Functional Polymers and Supramolecular Materials, 197921 National Sun Yat-Sen University, Kaohsiung 804, Taiwan; ‡ Department of Chemistry, Faculty of Science, Assiut University, Assiut 71515, Egypt; § Department of Medicinal and Applied Chemistry, Kaohsiung Medical University, Kaohsiung 807, Taiwan

**Keywords:** dye degradation, pyrene, conjugated microporous polymers, D-π-A structure, photocatalysis, thiazolothiazole

## Abstract

Water pollution caused by organic dyes poses a significant threat to ecosystems and human health, underscoring the urgent need for sustainable degradation methods. We report two donor−π–acceptor conjugated microporous polymers (CMPs), Pyr-Ph-TzTz and Pyr-Th-TzTz, assembled from pyrene (Pyr) donors, phenyl or thiophene π-bridges, and thiazolothiazole (TzTz) acceptors. Precursors [4,4′,4″,4‴-(pyrene-1,3,6,8-tetrayl)­tetrabenzaldehyde (Pyr-Ph-4CHO) and 5,5′,5″,5‴-(pyrene-1,3,6,8-tetrayl)­tetrakis­(thiophene-2-carbaldehyde) (Pyr-Th-4CHO)] were synthesized via electrophilic bromination and Suzuki–Miyaura coupling with 4-formylphenylboronic acid (PFPBA), and 5-formyl-2-thienylboronic acid (5-FTBA); respectively. Pyr-Ph-4CHO and Pyr-Th-4CHO were each subjected to a one-pot condensation reaction with dithiooxamide, yielding robust, thermally stable CMPs—Pyr-Ph-TzTz and Pyr-Th-TzTz—with amorphous frameworks and surface areas of 37 and 20 m^2 ^g^–1^, respectively. UV–vis spectra reveal narrow band gaps of 2.02 eV for Pyr-Ph-TzTz CMP and 2.39 eV for Pyr-Th-TzTz CMP. Pyr-Ph-TzTz CMP exhibits markedly enhanced charge separation, as evidenced by pronounced PL quenching and ultraviolet photoelectron spectroscopy (UPS) analysis. Both CMPs adsorb rhodamine B (RhB) rapidly (equilibrium in 30 min; 55% removal by Pyr-Ph-TzTz CMP, 90% by Pyr-Th-TzTz CMP) and degrade it under visible light, achieving 96% (*k* = 0.0545 min^–1^) and 39% (*k* = 0.00341 min^–1^) removal, respectively. Radical scavenging and EPR identify •OH as the primary active species. Remarkably, Pyr-Ph-TzTz CMP retains >90% activity after five cycles, highlighting its promise for solar-driven dye removal.

## Graphical abstract

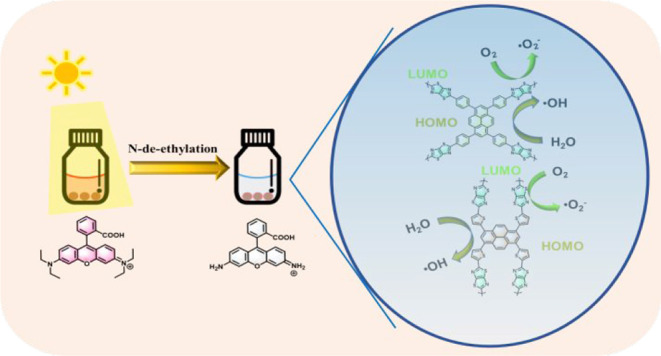

## 1 Introduction

With the rapid growth of the global population and accelerating industrialization, water pollution has emerged as a critical environmental challenge.^1–5^ Residual organic dyes in wastewater not only deepen water coloration, reduce light penetration, and inhibit the growth of aquatic plants and photosynthetic microorganisms but also readily coordinate with metal ions to form complex species whose toxicity poses micro- to acute-level threats to fish and other aquatic organisms.^6–8^ Consequently, developing efficient and environmentally friendly dye removal technologies is essential for safeguarding ecological balance and human health. Although physical adsorption, chemical oxidation–reduction, and biological degradation methods have been widely studied and implemented, many suffer from low treatment efficiencies, the potential for secondary pollution, or prohibitive operating costs.^9–12^ In contrast, photocatalysis harnesses solar energy, a clean, abundant, and nonpolluting resource, to activate semiconductor materials, generating electron–hole pairs that can drive the degradation of organic dyes under mild conditions. This approach has thus gained recognition as a promising green technology.^13–15^

Since the 1960s, the photoelectrochemical properties of semiconductors such as TiO_2_ and ZnO under ultraviolet irradiation have been systematically explored. In 1964, Honda and Fujishima first demonstrated the photosensitization effect of TiO_2_ electrodes, using a platinum cathode and a UV-illuminated TiO_2_ photoanode to split water into hydrogen and oxygen.^16,17^ Their pioneering work sparked extensive research into the photocatalytic decomposition of water and organic pollutants.^18–21^ Subsequent efforts have focused on novel photocatalysts, Bi_2_WO_6_,^22,23^ g-C_3_N_4_,^24,25^ and others,^26,27^ and on strategies such as doping,^28,29^ heterojunction construction,^30,31^ and surface modification^32,33^ to extend light absorption into the visible region and suppress charge recombination.^34–37^ However, the inherently wide bandgaps of many inorganic semiconductors limit their use to ultraviolet light, which accounts for only 3–5% of the solar spectrum, and they often suffer from rapid electron–hole recombination.^38,39^

Recently, organic microporous materials with extended π-conjugation have attracted significant attention for photocatalytic applications. CMPs offer large surface areas, excellent thermal and chemical stability, and highly tunable electronic structures.^40–49^ Through careful monomer design and assembly, CMPs can be engineered to modulate band structures, while their ordered pore networks facilitate mass transport and charge carrier migration, thereby enhancing photocatalytic activity.^50,51^ Nevertheless, CMPs with excessively large bandgaps remain restricted to ultraviolet absorption, diminishing their overall solar utilization efficiency. To overcome the limitations in visible-light responsiveness, it is crucial to develop a class of donor (D)–acceptor (A) CMPs that are both highly efficient and easy to recover. By incorporating tunable D–A units, these materials can significantly enhance their photoelectric and photocatalytic properties.^52^ The exciton-driven photocatalytic behavior of D–A CMPs originates from the rapid exciton dissociation at the donor–acceptor interface, facilitating efficient interaction with organic dye pollutants. Such architecture promotes broad light absorption, optimized band gaps, effective charge separation, and enhanced charge mobility, ultimately boosting the concentration of excited electrons in the acceptor domain.^53^ This study adopts a donor-π-acceptor (D-π-A) strategy to design a new class of CMPs. Pyrene (Pyr) units, known for their strong electron-donating ability and planar π-frameworks, serve as the donor core.^54–56^ Phenyl and thiophene moieties act as π-bridges to enable efficient intramolecular charge transfer,^57,58^ while the thiazolothiazole (TzTz) units act as strong electron acceptors, imparting the polymers with dual nitrogen and sulfur doping.^55,59^ This D-π-A architecture not only broadens the light-absorption range into the visible region but also enhances electron–hole separation through internal push–pull interactions, reducing recombination rates.^60–64^

Drawing from the above findings, this work reports the design and synthesis of two pyrene-based donor-π-acceptor (D-π-A) conjugated microporous polymers, Pyr-Ph-TzTz and Pyr-Th-TzTz. In these materials, pyrene units act as electron donors, while phenyl or thiophene moieties serve as π-bridges to facilitate intramolecular charge transfer. Thiazolothiazole (TzTz) units, on the other hand, function as strong electron acceptors. The CMPs were synthesized via a one-pot condensation reaction of Pyr-Ph-4CHO or Pyr-Th-4CHO with dithiooxamide under solvothermal conditions, producing amorphous, thermally stable, and microporous networks. This molecular design aims to broaden light absorption into the visible region and enhance electron–hole separation efficiency, thereby overcoming the limitations of conventional CMPs with large band gaps. Such a strategy is expected to improve photocatalytic performance in dye degradation applications, addressing critical challenges in sustainable water treatment. Importantly, the synthesized CMPs demonstrate excellent chemical and photocatalytic stability, as evidenced by their ability to maintain over 90% dye degradation efficiency after five consecutive catalytic cycles, highlighting their practical recyclability.

## 2 Experimental Section

### 2.1 Materials

4-Formylphenylboronic acid (PFPBA, 98%), 5-formyl-2-thienylboronic acid (5-FTBA, 98%), and dithiooxamide were obtained from Sigma-Aldrich, as were bromine solution (Br_2_, 99%), nitrobenzene (99%), and pyrene (98%). Potassium carbonate (K_2_CO_3_, ≥ 99.8%) was purchased from SHOWA, and tetrakis­(triphenylphosphine)palladium [Pd­(PPh_3_)_4_,98%] from Leyan. Silver nitrite (AgNO_3_, 99%) and *p*-benzoquinone (BQ, 98%) were supplied by Alfa Aesar. 1,4-dioxane (DO, 99.8%) and N,N-dimethylformamide (DMF) were purchased from Fisher Chemical, while 2-propanol (IPA, 99.9%) and ethylenediaminetetraacetic acid disodium salt dihydrate (EDTA-2Na, 99%) were obtained from DUKSAN. In our previous work, we successfully synthesized 1,3,6,8-tetrabromopyrene (Pyr-4Br), Pyr-Ph-4CHO, and Pyr-Th-4CHO with slight modifications to the established procedures.^65^

### 2.2 Synthesis of Pyr-Ph-TzTz CMP

In a typical synthesis of Pyr-Ph-TzTz CMP, a 50 mL Schlenk tube was charged under argon with dithiooxamide (0.39 g, 3.23 mmol) and Pyr-Ph-4CHO (0.50 g, 0.81 mmol). Dry nitrobenzene (35 mL) was added, and the reaction mixture was stirred at 140 °C for 180 h. After cooling to room temperature, the dark brown precipitate was collected by filtration and then purified by Soxhlet extraction using DMF at 180 °C for 72 h to remove unreacted monomer and low-molecular-weight byproducts. The polymer yielded the Pyr-Ph-TzTz CMP as a fine brown powder.

### 2.3 Synthesis of Pyr-Th-TzTz CMP

An analogous procedure was employed to prepare the Pyr-Th-TzTz CMP: in a separate 50 mL Schlenk tube, 0.37 g of dithiooxamide (3.11 mmol) and 0.50 g of Pyr-Th-4CHO (0.78 mmol) were combined with 25 mL of dry nitrobenzene. This mixture was likewise heated at 140 °C for 180 h. After cooling to room temperature, the resulting black solid was isolated by filtration and subjected to Soxhlet extraction with DMF at 180 °C for 72 h to afford the Pyr-Th-TzTz CMP as a uniform black powder.

## 3 Results and Discussion

### 3.1 Synthesis and Structural Characterization of Pyr-TzTz-Based CMPs

The Pyr-based precursors for CMP synthesis were prepared via sequential electrophilic bromination and Suzuki-Miyaura coupling reactions (Schemes S1–S3). First, Pyr-4Br was obtained by treating pyrene with Br_2_ under electrophilic aromatic substitution (S_E_Ar) conditions. The successful introduction of bromine atoms was evidenced by the appearance of the absorption bands at 3077 cm^–1^ and 672 cm^–1^, corresponding to sp^2^ C–H and C–Br stretching vibrations, respectively (Figure S1), and by mass spectrometry, which showed a molecular peak at *m*/*z* 517 ([Pyr-4Br + H]^+^) (Figure S2). Subsequently, Pyr-4Br was subjected to Suzuki-Miyaura cross-coupling with either PFPBA or 5-FTBA to yield the tetra-aldehyde intermediates Pyr-Ph-4CHO and Pyr-Th-4CHO (Schemes S2 and S3). FT-IR spectra of Pyr-Ph-4CHO exhibited strong absorption bands at 1741 cm^–1^ and 1697 cm^–1^, attributed to the C=O stretching modes of the benzaldehyde functionalities, and a band at 2849 cm^–1^ characteristic of the aldehyde C–H stretch. In contrast, Pyr-Th-4CHO showed analogous C=O absorptions at 1740 cm^–1^ and 1667 cm^–1^, along with an aldehyde C–H band at 2852 cm^–1^ (Figure S3). The structures of both intermediates were further confirmed by ^1^H NMR spectroscopy (Figures S4 and S5). In the spectrum of Pyr-Ph-4CHO, the aldehydic proton resonated at 10.16 ppm, while the aromatic protons of the pyrene core and the phenyl substituents appeared between 8.18 and 7.85 ppm. For Pyr-Th-4CHO, the aldehyde proton signal was observed at 10.03 ppm, and the combined pyrene and thiophene aromatic signals spanned 8.60 ppm to 7.53 ppm. Mass spectrometry showed molecular peaks at *m*/*z* 619 ([Pyr-Ph-4CHO + H]^+^) (Figure S6) and *m*/*z* 641 ([Pyr-Th-4CHO – H]^−^) (Figure S7). Together, these spectroscopic data unambiguously establish the successful synthesis and purity of the precursor monomers used for subsequent CMP formation. As illustrated in Scheme 1a–d, the thiazolothiazole (TzTz)-linked CMPs (Pyr-Ph-TzTz (Scheme 1b) and Pyr-Th-TzTz (Scheme 1d) were obtained via a one-pot condensation–oxidation–cyclization between the tetra-aldehyde precursors (Pyr-Ph-4CHO or Pyr-Th-4CHO) and dithiooxamide. The disappearance of the carbonyl bands and the concomitant emergence of C=N stretching vibrations in the FT-IR spectra (Figure 1a) confirmed successful conversion of the aldehyde groups into thiazolothiazole linkages. Specifically, Pyr-Ph-TzTz CMP exhibited new bands at 3029, 1658, and 890 cm^–1^, assignable to sp^2^ C–H, C=N, and C–S stretches, while Pyr-Th-TzTz CMP showed analogous peaks at 3060, 1659, and 873 cm^–1^.

**Scheme 1:**
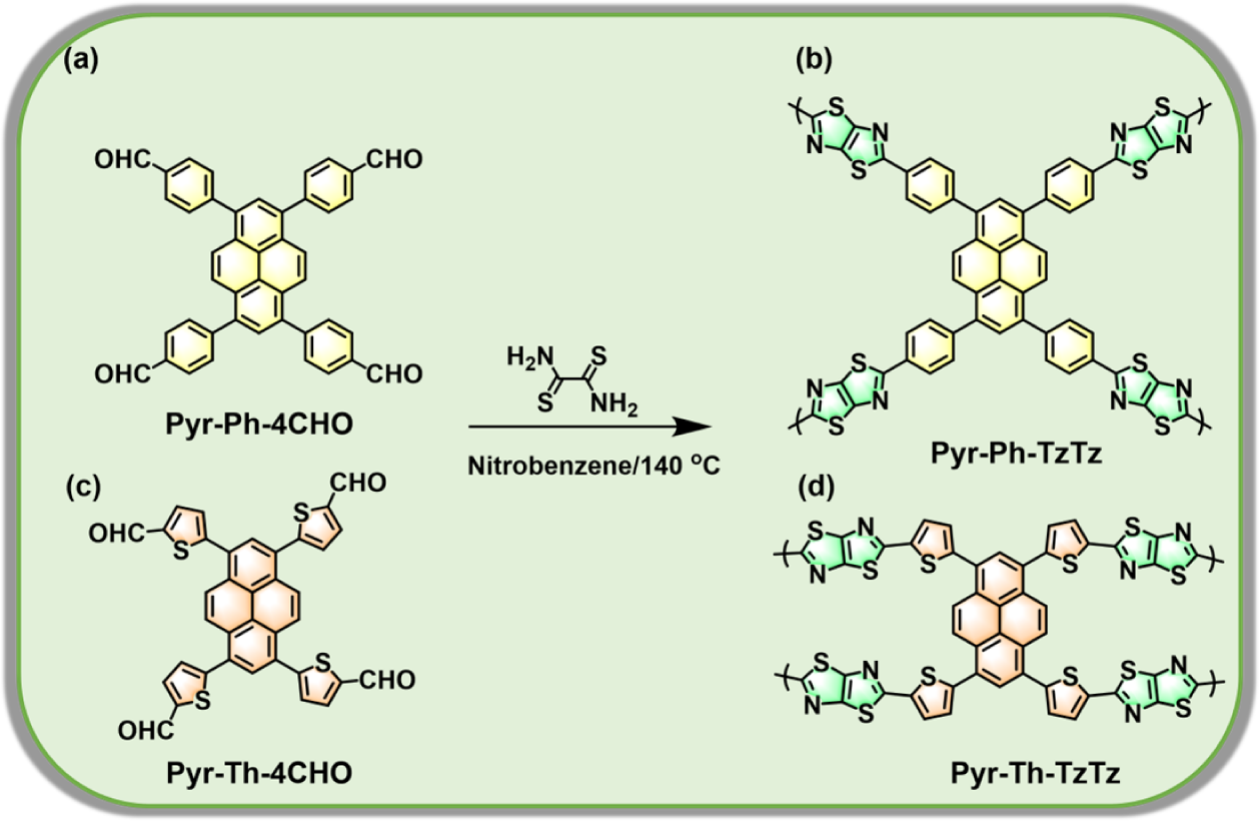
Synthesis of (b) Pyr-Ph-TzTz CMP and (d) Pyr-Th-TzTz CMP through the Reaction of (a) Pyr-Ph-4CHO and (c) Pyr-Th-4CHO with Dithiooxamide

**Figure 1:**
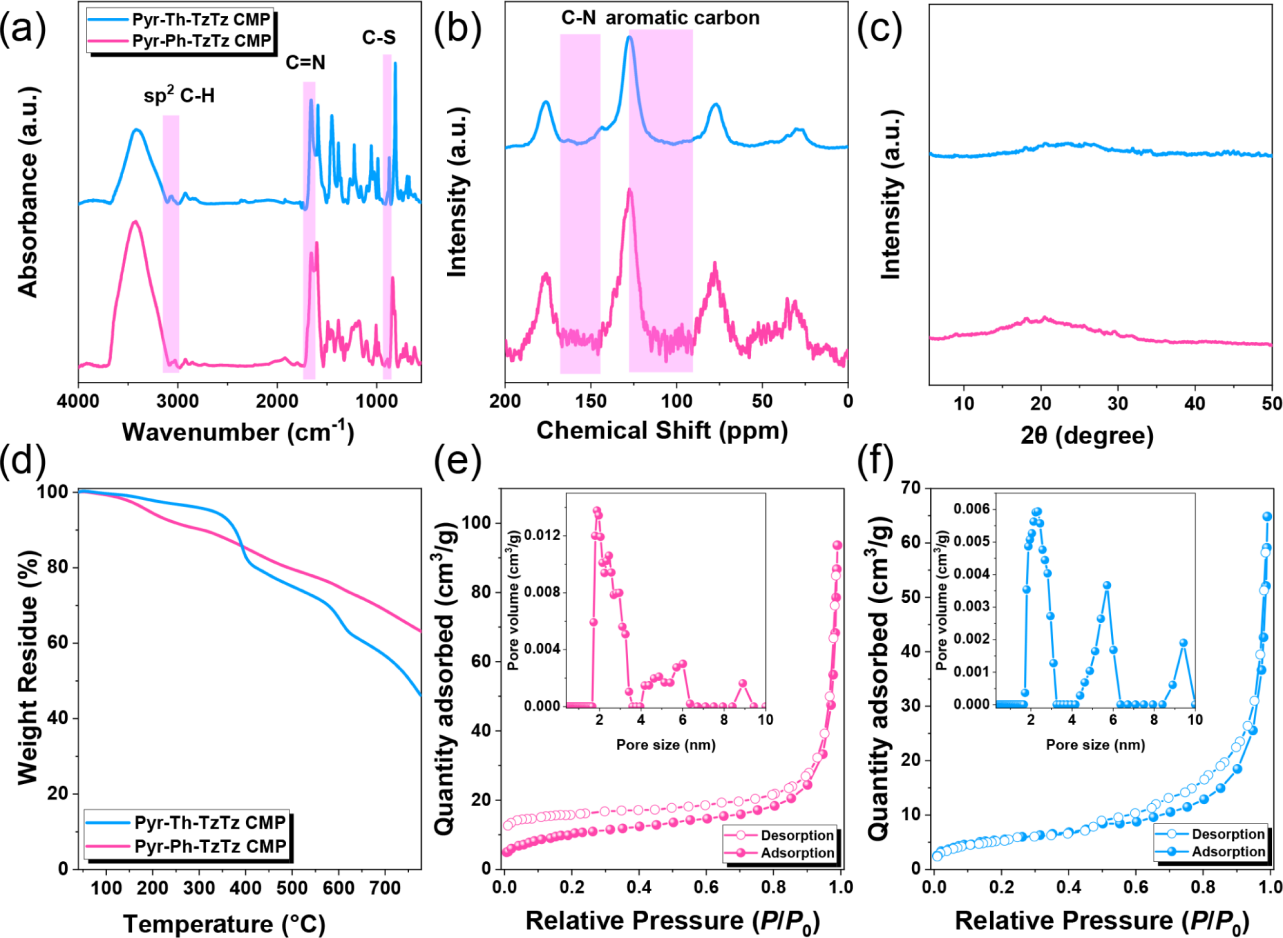
(a) FTIR spectra, (b) solid-state ^13^C NMR spectra, (c) XRD pattern, (d) TGA profile of Pyr-Ph-TzTz and Pyr-Th-TzTz CMPs. (e, f) N_2_ adsorption/desorption isotherms of (e) Pyr-Ph-TzTz and (f) Pyr-Th-TzTz CMPs (Inset Figure 1e,f; pore size distribution profiles of Pyr-Ph-TzTz and Pyr-Th-TzTz CMPs).

Solid-state ^13^C CP-MAS NMR spectra of both Pyr-Ph-TzTz and Pyr-Th-TzTz CMPs exhibit broad resonances between 175 and 127 ppm, attributable to the newly formed C=N linkages and the aromatic carbon structure in Figure 1b. Powder XRD patterns (Figure 1c) displayed only broad diffraction halos between 10° and 30°, indicating that both TzTz-linked CMPs possess an amorphous and disordered framework. Thermogravimetric analysis (TGA) (Figure 1d) demonstrated high thermal stability, with 10% weight loss temperatures (*T*_d10_) of 307 °C for Pyr-Ph-TzTz CMP and 377 °C for Pyr-Th-TzTz CMP, and residual char yields of 62% and 46%, respectively. Nitrogen adsorption–desorption isotherms measured at 77 K (Figure 1e) exhibited Type V behavior with similar H4 hysteresis loops, indicative of combined microporosity and mesoporosity. Brunauer–Emmett–Teller (BET) analysis yielded specific surface areas of 37 m^2^/g for Pyr-Ph-TzTz CMP and 20 m^2^/g for Pyr-Th-TzTz CMP. Pore size distributions calculated by nonlocal density functional theory (Figure 1f) confirmed a dominant micropore diameter of 1.8 nm for Pyr-Ph-TzTz CMP, whereas Pyr-Th-TzTz CMP featured a higher distribution centered at 2.3 nm (with additional mesopores at 5.7 and 9.4 nm).

High-resolution X-ray photoelectron spectroscopy (XPS) was employed to probe the local electronic environments of the TzTz-linked CMPs, with particular focus on the C 1s, N 1s, and S 2p regions (Figure 2a,e). In the C 1s spectrum of Pyr-Ph-TzTz CMP, three distinct components, corresponding to C–C/C=C, C=N/C=S, and C–N/C–S bonding, were centered at 283.10, 284.00, and 285.03 eV, respectively (Figure 2b). By contrast, incorporation of the thiophene spacer in Pyr-Th-TzTz CMP induced a systematic downshift of these signals to 282.80, 283.51, and 284.28 eV, reflecting an increase in electron density throughout the conjugated backbone (Figure 2f). A similar trend was observed in the N 1s region: the N–C and N=C peaks in Pyr-Ph-TzTz CMP appeared at 398.05 and 399.37 eV, whereas in Pyr-Th-TzTz CMP, they moved to lower binding energies of 397.56 and 398.68 eV (Figure 2c,g). Likewise, the sulfur core levels (S 2p_3/2_ and S 2p_1/2_) shifted from 162.50 and 163.80 eV in Pyr-Ph-TzTz CMP to 162.17 and 163.38 eV in the Pyr-Th-TzTz CMP (Figure 2d,h). These consistent binding-energy downshifts can be attributed to the electron-donating nature of the thiophene unit: its sulfur atom contributes additional lone-pair electron density into the extended π-system, thereby raising the local Fermi level and rendering all heteroatoms more electron-rich.

**Figure 2:**
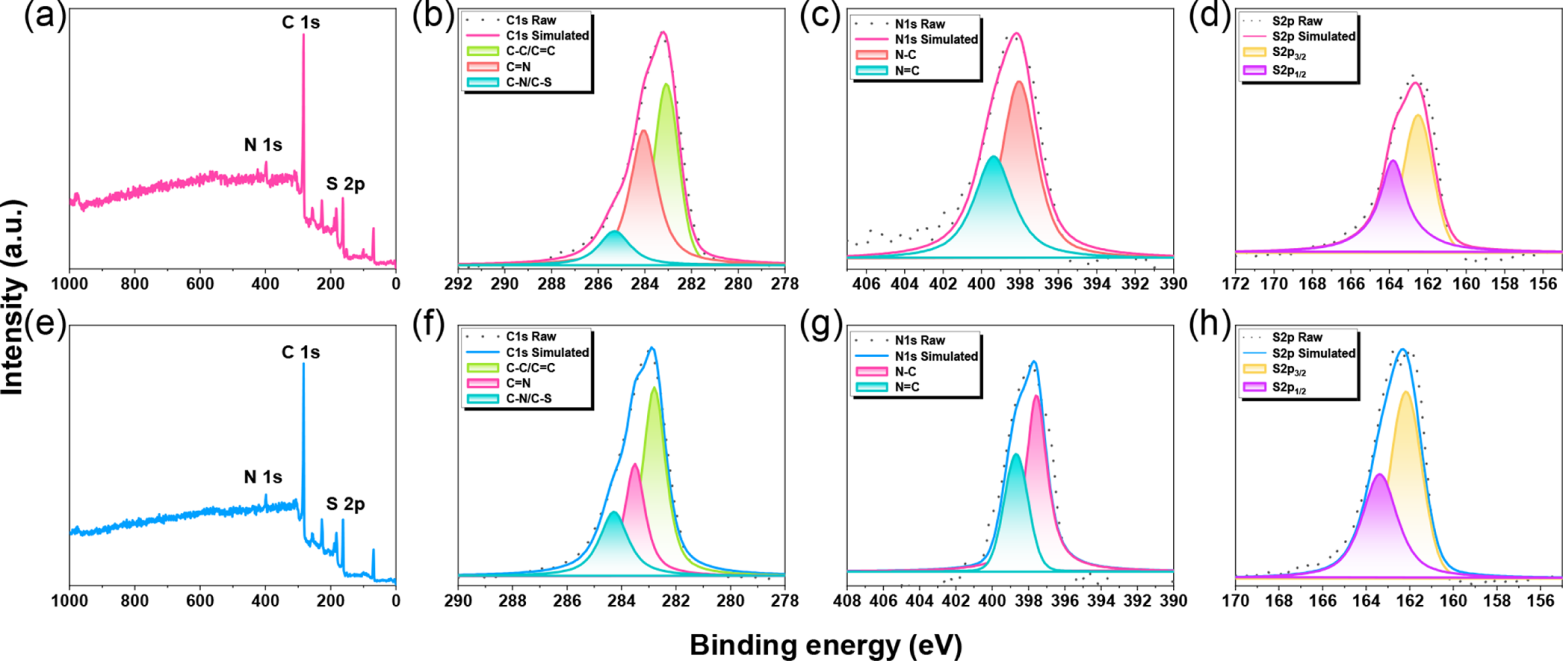
(a,e) XPS survey spectra and (b,c,d,f,g,h) deconvolution spectra of C 1s, N 1s, and S 2p scan profiles recorded for (a–d) Pyr-Ph-TzTz CMP and (e–h) Pyr-Th-TzTz CMPs.

This enhanced electron delocalization not only confirms the successful incorporation of the thiophene bridge but also suggests more facile charge transfer within the polymer matrix. This feature is expected to improve photocatalytic activity by reducing the potential barrier for photoinduced electron–hole separation. Scanning electron microscopy (SEM) coupled with energy-dispersive X-ray spectroscopy (EDS) was employed to elucidate the surface morphologies and elemental homogeneity of the two TzTz-linked CMPs. As shown in Figure 3a, Pyr-Ph-TzTz CMP forms dense, cauliflower-like aggregates composed of irregular nodules at 3000 magnifications. This compact architecture is indicative of a predominantly microporous network. EDS mapping of the same region (Figure 3b–d) confirms the uniform distribution of carbon, nitrogen, and sulfur throughout the sample, with quantitative atomic percentages detailed in Table S1. In contrast, Pyr-Th-TzTz CMP exhibits a loosely woven, fibrous morphology (Figure 3e), characterized by micrometer-long strands that interconnect to form open channels. Such a scaffold-like framework reflects the influence of the thiophene π-bridge on polymer packing, which favors more extended chain conformations and partial stacking, thereby creating a hierarchical micro/mesoporous network. EDS analysis likewise demonstrates a homogeneous dispersion of C, N, and S (Figure 3f–h), with elemental ratios comparable to those of Pyr-Ph-TzTz CMP (Table S1). Collectively, the combined SEM and SEM-EDS analyses highlight that the substitution of a phenyl (Ph) unit with a thiophene (Th) moiety not only modifies the macroscopic morphology of the CMP material but also maintains its compositional homogeneity. These structural and compositional features are believed to contribute to the increased surface area and porosity observed in Pyr-Th-TzTz CMP, which will be further examined in subsequent photodegradation and adsorption experiments.

**Figure 3:**
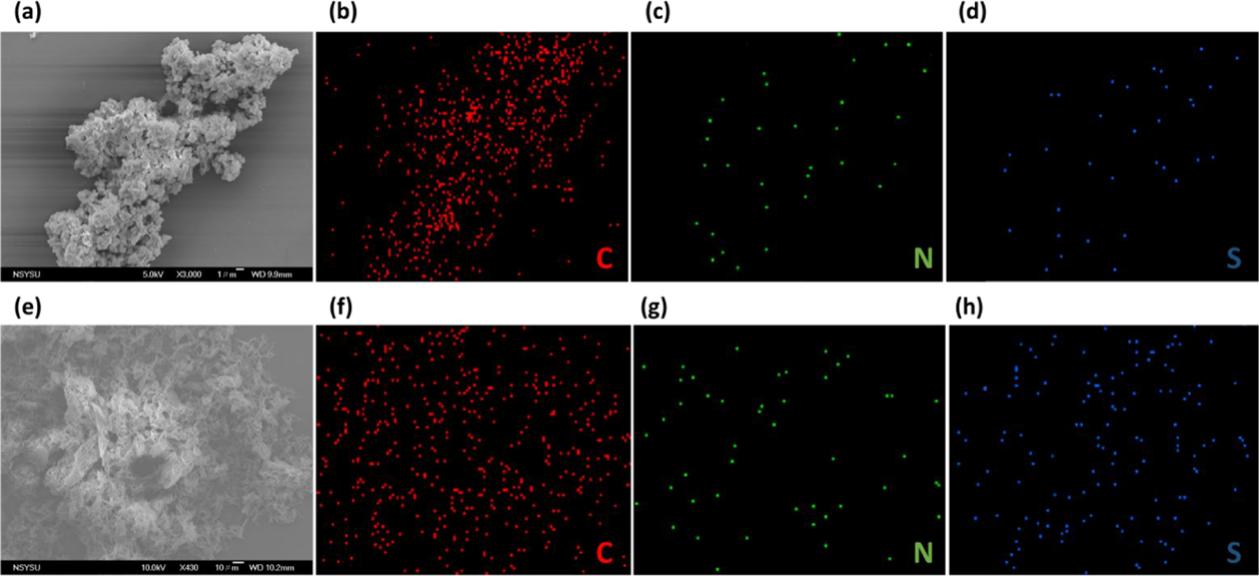
(a,e) SEM images and (b,c,d,f,g,h) SEM-EDS images of C, N, and S recorded for (a–d) Pyr-Ph-TzTz CMP and (e–h) Pyr-Th-TzTz CMPs.

### 3.2 Photophysical Properties and Adsorption Performance of Pyr-Ph-TzTz and Pyr-Th-TzTz CMPs

The photophysical properties of the two TzTz-linked CMPs, Pyr-Ph-TzTz and Pyr-Th-TzTz, were systematically investigated using UV–vis absorption spectroscopy, photoluminescence (PL) emission spectroscopy, and ultraviolet photoelectron spectroscopy (UPS). These techniques provided insight into their light absorption characteristics, band structures, and charge carrier dynamics, which are crucial for understanding their photocatalytic performance. As depicted in Figure 4a, Pyr-Ph-TzTz CMP exhibits absorption edges at approximately 460 and 508 nm in the visible region, whereas Pyr-Th-TzTz CMP extends its absorption from around 504 to 567 nm. The relatively red-shifted absorption of Pyr-Th-TzTz CMP indicates a broader visible-light harvesting capability than its phenylene-based analogue.^66^ Tauc analysis (Figure 4b) yields optical band gaps of 2.02 eV for Pyr-Ph-TzTz CMP and 2.39 eV for Pyr-Th-TzTz CMP; the smaller band gap of Pyr-Ph-TzTz CMP suggests that electronic transitions from the highest occupied molecular orbital (HOMO) to the lowest unoccupied molecular orbital (LUMO) occur with less energetic input, thereby potentially enhancing photocatalytic efficiency. PL emission spectra, recorded under excitation at 265 nm (Figure 4c), offer insight into charge carrier dynamics. Pyr-Ph-TzTz CMP shows significantly stronger PL quenching relative to Pyr-Th-TzTz CMP, implying more efficient separation of photogenerated electron–hole pairs in the former material. Since PL intensity is inversely correlated with carrier recombination rate, the lower emission from Pyr-Ph-TzTz CMP suggests reduced exciton recombination and, consequently, a higher likelihood of charge carriers participating in photocatalytic reactions.^67^ UPS measurements were employed to determine the HOMO energy levels of both CMPs. Figure 4d,e show the UPS spectra from which the secondary electron cutoff (*E*_cutoff_) and valence band onset (*E*_onset_) were extracted via linear extrapolation.^68^

**Figure 4:**
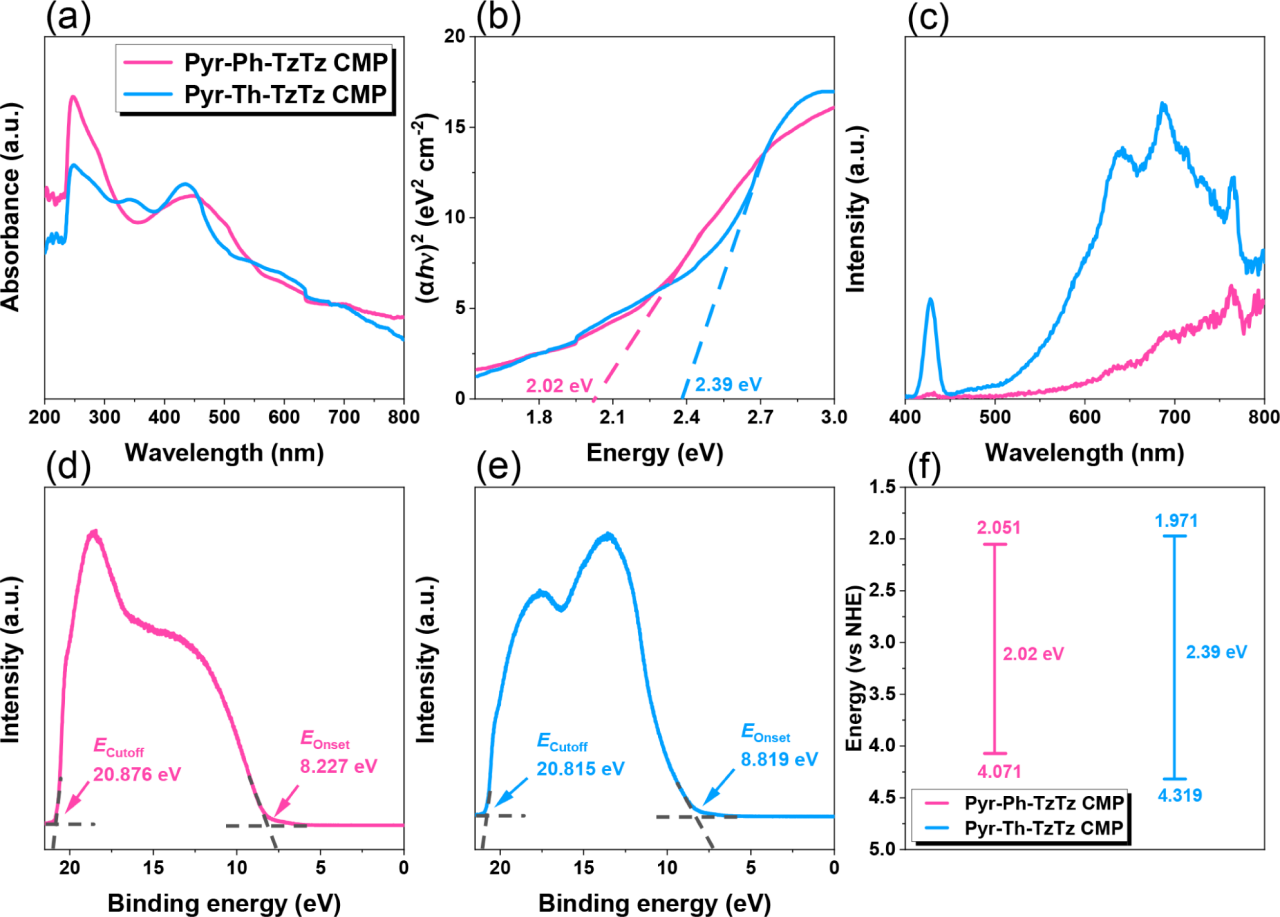
(a) UV–visible spectra, (b) Tauc plot, (c) PL spectra, (d,e) UPS spectra, and (f) energy level diagram of Pyr-Ph-TzTz and Pyr-Th-TzTz CMPs.

$$\varphi =h\nu -E_{\mathrm{cutoff}}\left(\mathrm{with}\;h\nu =21.22eV\;for\;He\;I\;excitation\right)$$

The work function φ was calculated for each sample. The HOMO levels under vacuum were then obtained from *–HOMO = φ + E*_*onset*_. These calculations yield HOMO energies of 4.071 eV for Pyr-Ph-TzTz CMP and 4.319 eV for Pyr-Th-TzTz CMP relative to the normal hydrogen electrode (NHE). When combined with the band gaps from the Tauc plots, these values allow the construction of the full band structure diagrams shown in Figure 4f. In summary, a narrower band gap of Pyr-Ph-TzTz CMP, stronger visible absorption, and more pronounced PL quenching, coupled with its favorable HOMO level, collectively point to superior photophysical properties for photocatalytic applications compared to Pyr-Th-TzTz CMP. To evaluate their photocatalytic potential, the synthesized CMPs were first assessed for their capacity to adsorb RhB, leveraging the π interactions between the aromatic and thiazolothiazole moieties as well as hydrogen bonding, as depicted in Schemes S4 and S5.^69,70^ Before and after adsorption in Figures S8 and S9, FTIR spectroscopy was employed to probe the interactions between the CMPs and RhB before and after adsorption. As shown in Figures S8 and S9, characteristic spectral changes were observed for both materials upon dye uptake. In the case of Pyr-Ph-TzTz CMP, a new C=O stretching vibration appeared at 1726 cm^–1^, and the C=N stretching band shifted from 1656 to 1664 cm^–1^. Similarly, Pyr-Th-TzTz CMP exhibited the emergence of a C=O band at 1739 cm^–1^ and a shift in the C=N signal from 1658 to 1655 cm^–1^ after adsorption. These observations confirm the presence of interactions between RhB and the structure of TzTz-linked CMP. Moreover, SEM images acquired after RhB adsorption show no discernible changes in morphology, indicating that the Pyr-Ph-TzTz and Pyr-Th-TzTz CMPs preserve their porous architecture and structural integrity during dye uptake and thus remain stable under the applied experimental conditions (Figures S10). For adsorption experiments, 5 mg of thiazolothiazole-linked CMPs were dispersed in 10 mL of 10 ppm RhB solution. UV–vis absorption spectra were recorded over a day to monitor dye uptake, as shown in Figure 5a,b. In both cases, the characteristic absorption peak of RhB at 554 nm gradually decreased with time, indicating successful adsorption. The Pyr-Ph-TzTz CMP achieved a removal efficiency of approximately 55%, while Pyr-Th-TzTz CMP reached 90% dye removal under identical conditions. These results align with literature reports, suggesting that the incorporation of heteroatoms such as nitrogen and sulfur can significantly enhance adsorption capacity due to increased interaction sites and electronic effects.^71–73^ To further investigate the thermodynamic behavior of the adsorption process, temperature-dependent studies were conducted at 25, 40, 60, and 80 °C. The corresponding UV–vis spectra (Figure S11) were analyzed using the Van’t Hoff equation, as plotted in Figure 5c. The negative slope of the Van’t Hoff plot indicates that the adsorption process is endothermic.^74^

**Figure 5:**
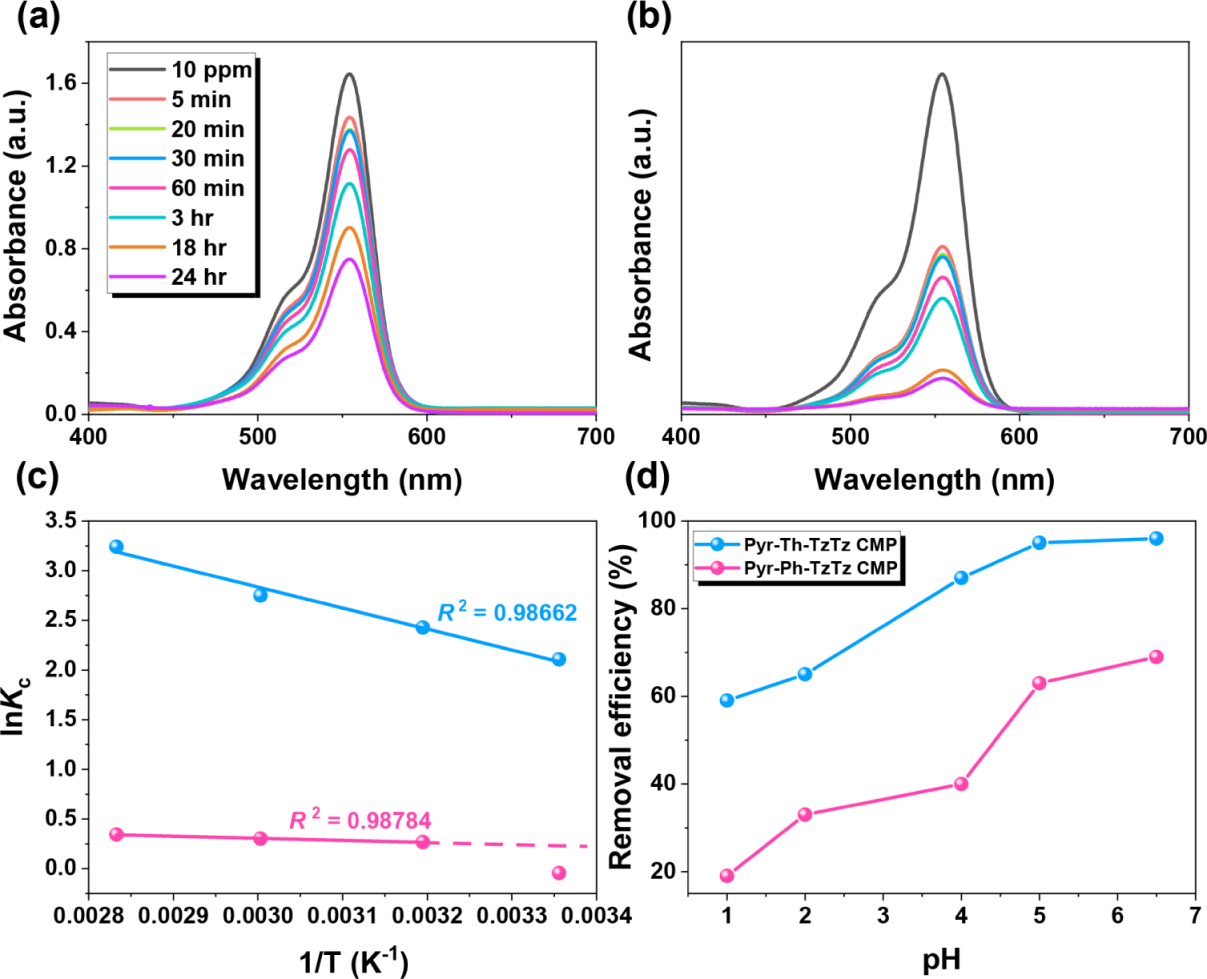
Effect of contact time on (a) Pyr-Ph-TzTz and (b) Pyr-Th-TzTz CMPs with 10 ppm RhB, (c) effect of different temperatures, and (d) effect of different pH values of TzTz-linked CMPs with 10 ppm RhB.

Thermodynamic parameters, including enthalpy change (Δ*H°*), entropy change (Δ*S°*), and Gibbs free energy change (Δ*G°*), were calculated using the equation as shown in eq 1:

1 $$\Delta G^{{}^{\circ}}=\Delta H^{{}^{\circ}}-T\Delta S^{{}^{\circ}}$$

The detailed numerical values of these parameters are summarized in Table S2. Since solution pH can influence the protonation state of both the cationic dye and nitrogen sites of thiazolothiazole-linked CMPs, the effect of pH on adsorption was also investigated. UV–vis spectra collected at pH values of 1, 3, 5, and 7 (Figure S12) show progressively stronger RhB uptake as pH increases (Figure 5d). At low pH, the protonation of the thiazolothiazole nitrogen leads to electrostatic repulsion with the cationic RhB (Scheme S6), thereby reducing adsorption. As the solution becomes less acidic, the polymers carry fewer positive charges, minimizing repulsion and allowing π-π stacking and hydrogen bonding to dominate, thus enhancing adsorption efficiency. The adsorption data were analyzed using both Langmuir and Freundlich isotherm models, as shown in Figure S13. The Langmuir isotherm model demonstrated a significantly better fit to the experimental data, with correlation coefficients (*R* ^2^) of 0.9282 and 0.9325, compared to the Freundlich model, which showed lower *R* ^2^ values of 0.6375 and 0.3566. Given that the Langmuir model is based on the assumption of monolayer adsorption on a homogeneous surface, these results indicate that the adsorption process in this study is primarily governed by monolayer coverage on uniform adsorption sites.^73^ Table S3 summarizes the maximum adsorption capacities of Pyr-Ph-TzTz CMP and Pyr-Th-TzTz CMP at different RhB concentrations. Across three consecutive cycling tests, the Pyr-Ph-TzTz and Pyr-Th-TzTz CMPs preserved their physicochemical properties and morphology with minimal change, underscoring their structural integrity and stability during repeated adsorption processes (Figure S14).

### 3.3 Degradation Performance of Pyr-Ph-TzTz and Pyr-Th-TzTz CMPs

To evaluate the intrinsic stability of RhB under visible-light irradiation, a control experiment was first conducted in the absence of any photocatalyst. A 30 ppm RhB solution was exposed to visible light for 60 min, and UV–vis measurements (Figure S15) confirmed that the characteristic absorption peak at 554 nm remained essentially unchanged, indicating negligible self-degradation of the dye under these conditions. Next, to eliminate any contribution from dye adsorption during photocatalytic testing, each CMP was first dispersed in the RhB solution and stirred in the dark until the adsorption–desorption equilibrium was reached. As shown in Figure S16, both TzTz-linked CMPs attained equilibrium within approximately 30 min, ensuring that subsequent changes in dye concentration under illumination reflect true photocatalytic degradation rather than further adsorption phenomena. Photocatalytic degradation experiments were then performed by adding 10 mg of each CMP to 20 mL of 30 ppm RhB and monitoring the color change under visible-light illumination (Figure 6a,b).

**Figure 6:**
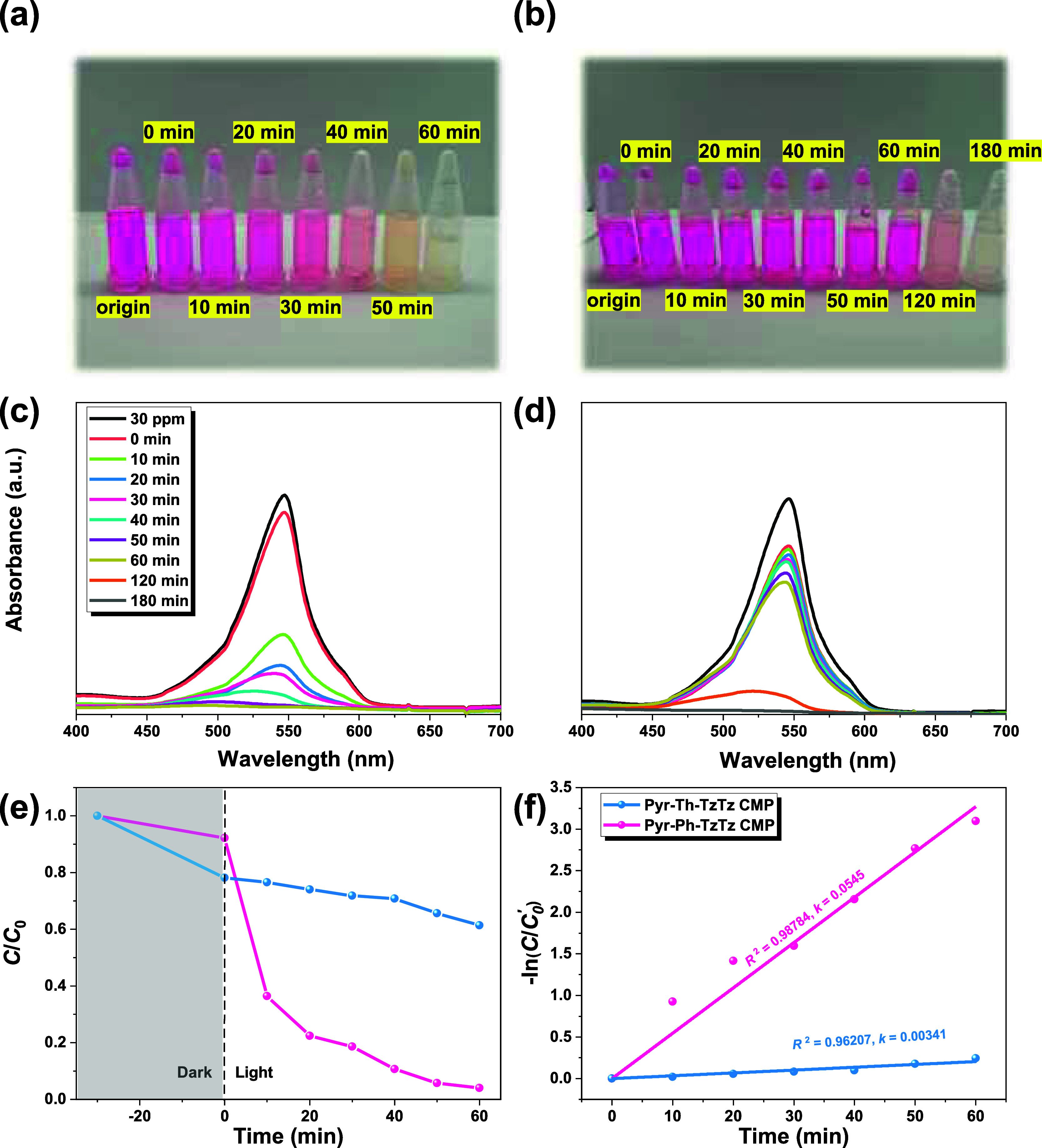
Photos of RhB solution after 60 min of photocatalytic degradation with (a) Pyr-Ph-TzTz and (b) Pyr-Th-TzTz CMPs, UV–vis spectra of RhB degradation with (c) Pyr-Ph-TzTz and (d) Pyr-Th-TzTz CMPs, and (e and f) plots of *C*/*C*_0_ and ln­(C/*C*_0_′) against irradiation time for TzTz-linked CMPs.

In the dark (Figure S17), Pyr-Th-TzTz CMP again outperformed Pyr-Ph-TzTz CMP in adsorption, but upon light irradiation (Figure 6c,d), the main RhB absorption peak red-shifted from 547 nm to ∼490 nm, consistent with N-de-ethylation and subsequent chromophore breakdown via radical attack.

Concomitantly, Pyr-Ph-TzTz CMP exhibited markedly superior photocatalytic activity: after 60 min, Pyr-Ph-TzTz CMP degraded ∼96% of RhB, whereas Pyr-Th-TzTz CMP degraded only ∼39% (Figure 6 e). Kinetic analysis employed the pseudo-first-order model as shown in eq 2:

2 $$\ln\left(C_{0}^{\prime }/C_{t}\right)=kt$$

where *C*_0_′ is the pollutant concentration at the onset of light irradiation in 0 min, *C*_t_ is the concentration at time *t*, and *k* is the rate constant. Linear fits (Figure 6f) yielded *k* = 0.0545 min^–1^ for Pyr-Ph-TzTz CMP (*R* ^2^ = 0.98784) and *k* = 0.00341 min^–1^ for Pyr-Th-TzTz CMP (*R* ^2^ = 0.96207), reinforcing the superior photodegradation efficiency of Pyr-Ph-TzTz CMP. Liquid chromatography–mass spectrometry (LC-MS) analysis of the reaction mixture at various irradiation intervals (Scheme S7, Figure S17) confirmed stepwise RhB fragmentation (*m*/*z* 443) into smaller species (*m*/*z* 331, 317, 273, 73, 60), corroborating a radical-driven oxidative cleavage mechanism on the surface of the Pyr-Ph-TzTz CMP.

The photocatalytic durability of Pyr-Ph-TzTz CMP was assessed via repeated degradation cycles of RhB under visible-light irradiation. As shown in Figure 7a, after five sequential runs, the degradation efficiency declined only marginally from 97% to 91%, underscoring the material’s excellent stability and reusability. To elucidate the charge separation and reactive species involved in the photocatalytic process, scavenger experiments were conducted (Figure 7b). Addition of AgNO_3_ to quench photogenerated electrons (e^–^) reduced the degradation efficiency from 97.2% (control group) to 94.3%, whereas IPA, a hydroxyl radical (•OH) scavenger, and EDTA-2Na, a hole (h^+^) scavenger, led to more pronounced decreases to 50.5% and 55.2%, respectively. BQ, which captures superoxide radicals (•O_2_^–^), only slightly suppressed the reaction (94.3%), indicating that •OH and h^+^ play dominant roles in RhB degradation, followed by e^–^ and •O_2_^–^. Electron paramagnetic resonance (EPR) spectroscopy with 5,5-dimethyl-1-pyrroline N-oxide (DMPO) as the spin trap provided further confirmation of radical generation (Figure S18). Under dark conditions, no DMPO–OH signal was detected; upon light exposure, a characteristic 1:2:2:1 quartet attributed to the DMPO–OH adduct emerged and intensified over time, demonstrating that visible-light irradiation stimulates •OH formation on the surface of Pyr-Ph-TzTz CMP.^75^

**Figure 7:**
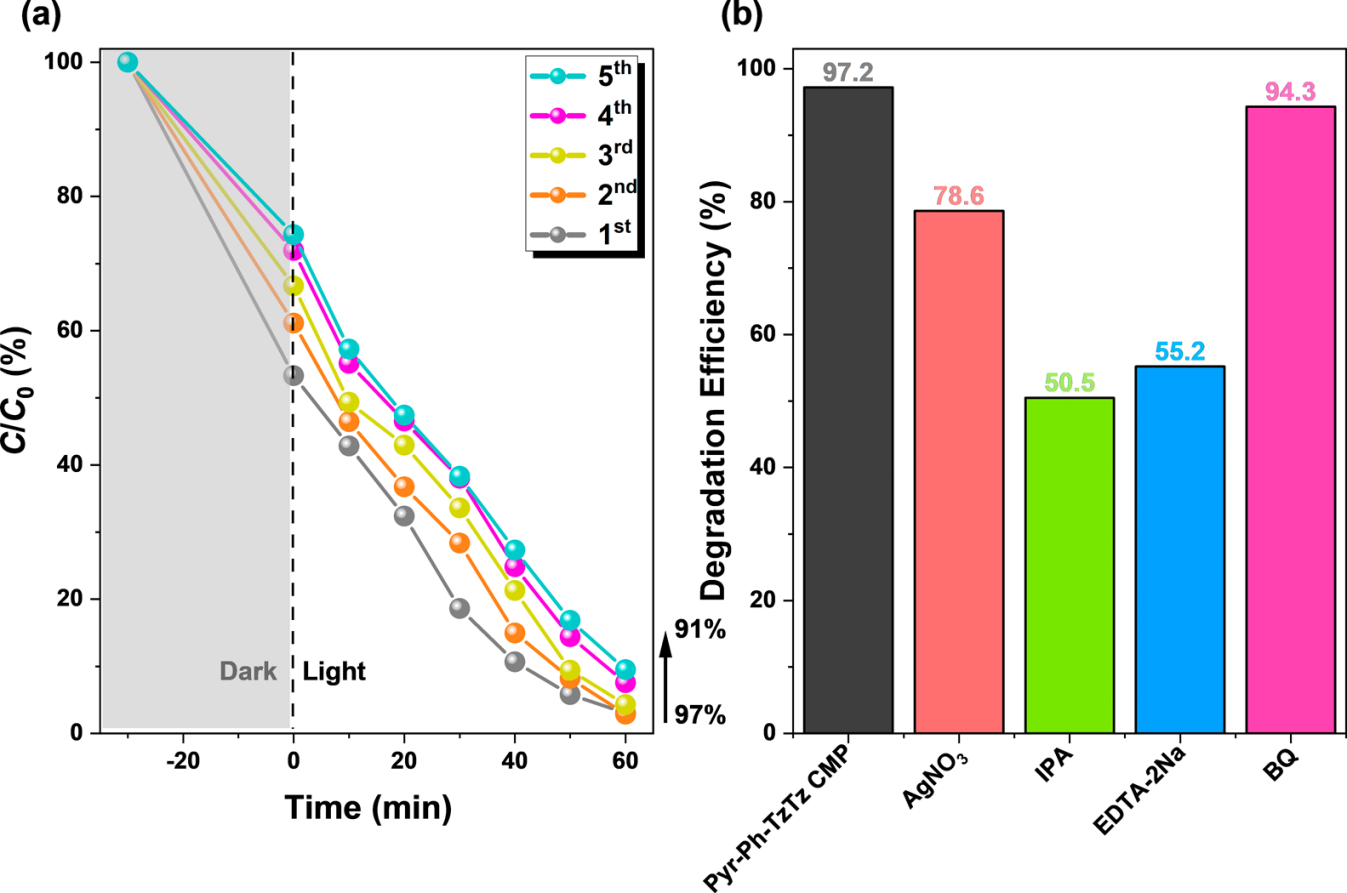
(a) The cyclic photodegradation of the RhB solution with Pyr-Ph-TzTz CMP, (b) experiments with various scavengers for the photodegradation of the RhB solution of Pyr-Ph-TzTz CMP.

Based on these findings, the proposed photocatalytic mechanism (Figure 8) involves photoexcitation of electrons from the highest occupied molecular orbital (HOMO) to the lowest unoccupied molecular orbital (LUMO) upon visible-light absorption. The photogenerated holes oxidize water or surface hydroxyl groups to yield •OH according to the equations below:

$$H_{2}O+h^{+}\to H^{+}+•OH$$

while the excited electrons reduce molecular oxygen to superoxide radicals:

$$e^{-}+O_{2}\to •{O_{2}}^{-}$$

$$•{O_{2}}^{-}+e^{-}+2H^{+}\to H_{2}O_{2}$$

$$H_{2}O_{2}+e^{-}\to •OH+OH^{-}$$

**Figure 8:**
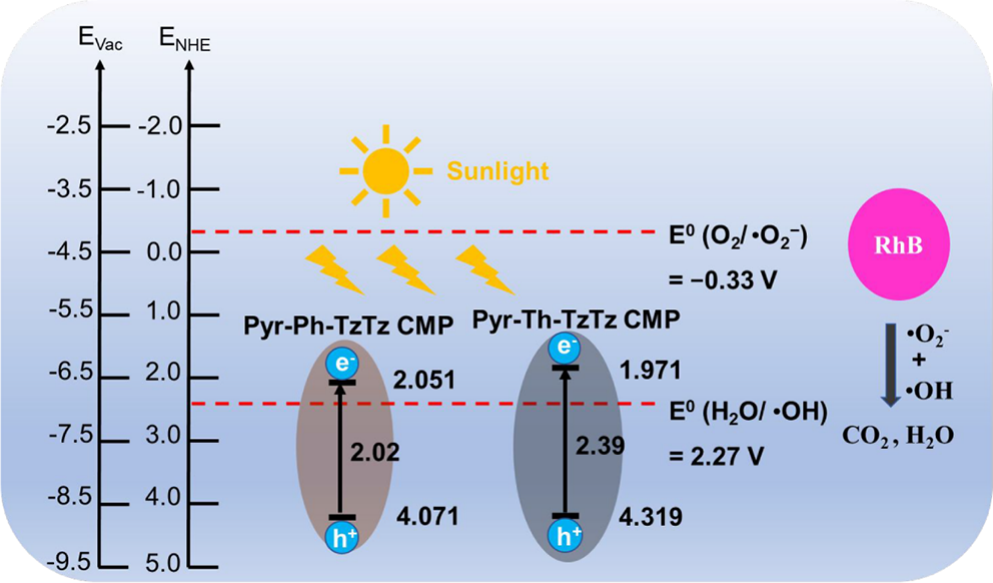
Photocatalytic reaction illustration of Pyr-Ph-TzTz CMP and Pyr-Th-TzTz CMP toward RhB solution under irradiation.

These reactive oxygen species attack RhB, ultimately mineralizing it to CO_2_ and H_2_O. The preeminence of •OH radicals, corroborated by both scavenger tests and EPR data, explains the high photocatalytic efficiency of Pyr-Ph-TzTz CMP.

## 4 Conclusions

In this study, we successfully developed two novel donor−π–acceptor (D-π-A) conjugated microporous polymers (CMPs), Pyr-Ph-TzTz and Pyr-Th-TzTz, featuring pyrene donor cores, phenyl or thiophene π-bridges, and thiazolothiazole acceptor units. This rational molecular design enables efficient tuning of the electronic structure, resulting in enhanced visible-light absorption and improved charge carrier separation. The incorporation of thiophene as a π-bridge further modulates the porosity and electron density, offering a unique mixed micro/mesoporous framework. Among the two, Pyr-Ph-TzTz CMP demonstrated superior photocatalytic performance and remarkable stability, maintaining high activity over multiple reuse cycles. These findings highlight the potential of D-π-A structured CMPs as a versatile platform for designing high-performance, stable, and recyclable photocatalysts. By integrating tailored molecular architecture with porous frameworks, this work provides valuable insights for advancing sustainable photocatalytic materials aimed at efficient organic pollutant degradation under visible light. The strategy presented herein can inspire future developments in the design of functional porous polymers for environmental remediation and energy applications.

## Supplementary Material



## Notes

Characterization, adsorption and degradation kinetics, photocatalytic experiments, schematic representations of the monomer preparation process along with their FTIR and NMR spectra, and schematic illustrations of the possible RhB adsorption mechanisms for Pyr-Ph-TzTz CMP and Pyr-Th-TzTz CMP, stability data of Pyr-Ph-TzTz CMP and Pyr-Th-TzTz CMP before and after RhB adsorption UV–vis spectra Pyr-Ph-TzTz CMP and Pyr-Th-TzTz CMP at different temperatures with 10 ppm RhB solution and different pH with 10 ppm RhB solution, Langmuir fitting and Freundlich fitting of thiazolothiazole-linked CMPs, UV spectra of Rhodamine B dye under visible light irradiation for different times, LC-MS analysis of photodegradation of RhB over Pyr-Ph-TzTz CMP under light irradiation (PDF)

## References

[1] Parvin F., Islam S., Akm S. I., Urmy Z., Ahmed S. (2020). A study on
the solutions of environment pollutions and worker’s health
problems caused by textile manufacturing operations. Biomed. J. Sci. Technol. Res..

[2] Mohamed M. G., Elewa A. M., Chen N. P., Mohammed A. A. K., Kuo S. W. (2025). Construction
of malononitrile-functionalized conjugated microporous polymers as
adsorbents for effective adsorption of Rhodamine B and density functional
theory perspective. Colloids Surf. A: Physicochem.
Eng. Asp..

[3] Arif A., Malik M. F., Liaqat S., Aslam A., Mumtaz K., Afzal A., Ch D. M., Nisa K., Khurshid F., Arif F. (2020). Water pollution and
industries. Pure Appl.
Biol..

[4] Hsiao C. W., Elewa A. M., Mohamed M. G., Kotp M. G., Chou M. M. C., Kuo S. W. (2024). Designing strategically functionalized hybrid porous
polymers with octavinylsilsesquioxane/dibenzo­[g,p]­chrysene/benzo­[c]-1,2,5-thiadiazole
units for rapid removal of Rhodamine B dye from water. Colloids Surf. A: Physicochem. Eng. Asp..

[5] Hsiao C. W., Elewa A. M., Mohamed M. G., Kuo S. W. (2024). Highly stable hybrid
porous polymers containing polyhedral oligomeric silsesquioxane (POSS)/Dibenzo­[g,p]­chrysene
and Dibenzo­[b,d]­thiophene units for efficient Rhodamine B dye removal. Sep. Purif. Technol..

[6] Robinson T., McMullan G., Marchant R., Nigam P. (2001). Remediation of dyes
in textile effluent: a critical review on current treatment technologies
with a proposed alternative. Bioresour. Technol..

[7] Al-Tohamy R., Ali S. S., Li F., Okasha K. M., Mahmoud Y. A. G., Elsamahy T., Jiao H., Fu Y., Sun J. (2022). A critical
review on the treatment of dye-containing wastewater: Ecotoxicological
and health concerns of textile dyes and possible remediation approaches
for environmental safety. Ecotoxicol. Environ.
Saf..

[8] Tkaczyk A., Mitrowska K., Posyniak A. (2020). Synthetic organic dyes as contaminants
of the aquatic environment and their implications for ecosystems:
A review. Sci. Total Environ..

[9] Katheresan V., Kansedo J., Lau S. Y. (2018). Efficiency
of various recent wastewater
dye removal methods: A review. J. Environ. Chem.
Eng..

[10] Kotp M.G., Mohamed M.G., Wang P.T., Hassan A.E., Elewa A.M., Kuo S.W. (2025). Unlocking the Potential of N,N,N′,N′-Tetraphenylbenzidine
Based on Conjugated Microporous Polymers for Rhodamine B Adsorption:
A Synergistic Experimental and Density Functional Theory Perspective. ACS Polym. Au.

[11] Lan D., Zhu H., Zhang J., Li S., Chen Q., Wang C., Wu T., Xu M. (2022). Adsorptive
removal of organic dyes via porous materials
for wastewater treatment in recent decades: A review on species, mechanisms
and perspectives. Chemosphere.

[12] Nachiyar C. V., Rakshi A., Sandhya S., Jebasta N. B. D., Nellore J. (2023). Developments
in treatment technologies of dye-containing effluent: a review. Case Stud. Chem. Environ. Eng..

[13] Saad I., Ralha N., Abukhadra M. R., Al Zoubi W., Ko Y. G. (2023). Recent
advances in photocatalytic oxidation techniques for decontamination
of water. J. Water Process. Eng..

[14] Chang S. Y., Elewa A. M., Mohamed M. G., Mekhemer I. M. A., Samy M. M., Zhang K., Chou H. H., Kuo S. W. (2023). Rational design
and synthesis of bifunctional Dibenzo [g,p] chrysene-based conjugated
microporous polymers for energy storage and visible light-driven photocatalytic
hydrogen evolution. Mater. Today Chem..

[15] Samarasinghe L. V., Muthukumaran S., Baskaran K. (2024). Recent advances in visible light-activated
photocatalysts for degradation of dyes: A comprehensive review. Chemosphere.

[16] Schneider J., Matsuoka M., Takeuchi M., Zhang J., Horiuchi Y., Anpo M., Bahnemann D. W. (2014). Understanding TiO_2_ photocatalysis:
mechanisms and materials. Chem. Rev..

[17] Banerjee A. N. (2011). The design,
fabrication, and photocatalytic utility of nanostructured semiconductors:
focus on TiO_2_-based nanostructures. Nanotechnol., Sci. Appl..

[18] Saeed M., Muneer M., Haq A. U., Akram N. (2022). Photocatalysis: An
effective tool for photodegradation of dyes—A review. Environ. Sci. Pollut. Res..

[19] Chiu Y. H., Chang T. F. M., Chen C. Y., Sone M., Hsu Y. J. (2019). Mechanistic
insights into photodegradation of organic dyes using heterostructure
photocatalysts. Catalysts.

[20] Anwer H., Mahmood A., Lee J., Kim K. H., Park J. W., Yip A. C. (2019). Photocatalysts for degradation of dyes in industrial
effluents: Opportunities and challenges. Nano
Res..

[21] Ajmal A., Majeed I., Malik R. N., Idriss H., Nadeem M. A. (2014). Principles
and mechanisms of photocatalytic dye degradation on TiO_2_ based photocatalysts: a comparative overview. RSC Adv..

[22] Chen T., Liu L., Hu C., Huang H. (2021). Recent advances on Bi_2_WO_6_-based photocatalysts
for environmental and energy
applications. Chin. J. Catal..

[23] Shang M., Wang W., Sun S., Zhou L., Zhang L. (2008). Bi_2_WO_6_ nanocrystals
with high photocatalytic activities under
visible light. J. Phys. Chem. C.

[24] Muhmood T., Ahmad I., Haider Z., Haider S. K., Shahzadi N., Aftab A., Ahmed S., Ahmad F. (2024). Graphene-like graphitic
carbon nitride (g-C_3_N_4_) as a semiconductor photocatalyst:
Properties, classification, and defects engineering approaches. Mater. Today. Sustain..

[25] Hassan A. E., Elsayed M. H., Hussien M. S. A., Mohamed M. G., Kuo S. W., Chou H. H., Yahia I. S., Mohamed T. A., Wen Z. (2023). V_2_O_5_ nanoribbons/N-deficient g-C_3_N_4_ heterostructure for enhanced visible-light photocatalytic performance. Int. J. Hydrogen Energy.

[26] Guo Q. Z., Zhou C., Ma Z. X., Yang X. (2019). Fundamentals of TiO_2_ photocatalysis: concepts, mechanisms,
and challenges. Adv. Mater..

[27] Lee K. M., Lai C. W., Ngai K. S., Juan J. C. (2016). Recent developments
of zinc oxide based photocatalyst in water treatment technology: a
review. Water Res..

[28] Kanakaraju D., Anak Kutiang F. D., Lim Y. C., Goh P. S. (2022). Recent progress
of Ag/TiO_2_ photocatalyst for wastewater treatment: Doping,
co-doping, and green materials functionalization. Appl. Mater. Today.

[29] Fang W., Yan J., Wei Z., Liu J., Guo W., Jiang Z., Shangguan W. (2024). Account of doping photocatalyst for water splitting. Chin. J. Catal..

[30] Acharya R., Parida K. (2020). A review on TiO_2_/g-C_3_N_4_ visible-light-responsive photocatalysts for
sustainable energy generation
and environmental remediation. J. Environ. Chem.
Eng..

[31] Liu J., Cheng B., Yu J. (2016). A new understanding
of the photocatalytic
mechanism of the direct Z-scheme g-C_3_N_4_/TiO_2_ heterostructure. Phys. Chem. Chem.
Phys..

[32] Zhao W., Adeel M., Zhang P., Zhou P., Huang L., Zhao Y., Ahmad M. A., Shakoor N., Lou B., Jiang Y. (2022). A critical
review on surface-modified nano-catalyst
application for the photocatalytic degradation of volatile organic
compounds. Environ. Sci.: Nano.

[33] Feng C., Wu Z. P., Huang K. W., Ye J., Zhang H. (2022). Surface modification
of 2D photocatalysts for solar energy conversion. Adv. Mater..

[34] Chong M. N., Jin B., Chow C. W., Saint C. (2010). Recent developments in photocatalytic
water treatment technology: a review. Water
Res..

[35] Li X., Chen Y., Tao Y., Shen L., Xu Z., Bian Z., Li H. (2022). Challenges
of photocatalysis and
their coping strategies. Chem. Catal..

[36] Zhu D., Zhou Q. (2019). Action and mechanism
of semiconductor photocatalysis on degradation
of organic pollutants in water treatment: A review. Environ. Nanotechnol., Monit. Manage..

[37] Karthikeyan C., Arunachalam P., Ramachandran K., Al-Mayouf A. M., Karuppuchamy S. (2020). Recent advances in semiconductor
metal oxides with
enhanced methods for solar photocatalytic applications. J. Alloys Compd..

[38] Horikoshi S., Saitou A., Hidaka H., Serpone N. (2003). Environmental remediation
by an integrated microwave/UV illumination method. V. Thermal and
nonthermal effects of microwave radiation on the photocatalyst and
on the photodegradation of rhodamine-B under UV/Vis radiation. Environ. Sci. Technol..

[39] Bera S., Won D. I., Rawal S. B., Kang H. J., Lee W. I. (2019). Design
of visible-light photocatalysts by coupling of inorganic semiconductors. Catal. Today.

[40] Mohamed M. G., Chen C. C., Ibrahim M., Mousa A. O., Elsayed M. H., Ye Y., Kuo S. W. (2024). Tetraphenylanthraquinone
and Dihydroxybenzene-Tethered
Conjugated Microporous Polymer for Enhanced CO_2_ Uptake
and Supercapacitive Energy Storage. JACS Au.

[41] Samy M. M., Mohamed M. G., Sharma S. U., Chaganti S. V., Lee J. T., Kuo S. W. (2024). An Ultrastable Tetrabenzonaphthalene-Linked
conjugated
microporous polymer functioning as a high-performance electrode for
supercapacitors. J. Taiwan Inst. Chem. Eng..

[42] Mohamed M. G., EL-Mahdy A. F. M., Kotp M. G., Kuo S. W. (2022). Advances in porous
organic polymers: syntheses, structures, and diverse applications. Mater. Adv..

[43] Mohamed M. G., Atayde E. C., Matsagar B. M., Na J., Yamauchi Y., Wu K. C.-W., Kuo S. W. (2020). Construction Hierarchically
Mesoporous/Microporous Materials Based on Block Copolymer and Covalent
Organic Framework. J. Taiwan Inst. Chem. Eng..

[44] Mohamed M. G., Sharma S. U., Wang P. T., Ibrahim M., Lin M. H., Liu C. L., Ejaz M., Yen H. J., Kuo S. W. (2024). Construction
of fully π-conjugated, diyne-linked conjugated microporous polymers
based on tetraphenylethene and dibenzo [g, p] chrysene units for energy
storage. Polym. Chem..

[45] Mohamed M. G., Chen T. C., Kuo S. W. (2021). Solid-state
chemical transformations
to enhance gas capture in benzoxazine-linked conjugated microporous
polymers. Macromolecules.

[46] Mohamed M. G., Chang W. C., Kuo S. W. (2022). Crown ether-and
benzoxazine-linked
porous organic polymers displaying enhanced metal ion and CO_2_ capture through solid-state chemical transformation. Macromolecules.

[47] Mohamed M. G., Mansoure T. H., Samy M. M., Takashi Y., Mohammed A. A., Ahamad T., Alshehri S. M., Kim J., Matsagar B. M., Wu K. C. W., Kuo S.-W. (2022). Ultrastable conjugated
microporous
polymers containing benzobisthiadiazole and pyrene building blocks
for energy storage applications. Molecules.

[48] Ma H., Chen Y., Li X., Li B. (2021). Advanced applications
and challenges of electropolymerized conjugated microporous polymer
films. Adv. Funct. Mater.

[49] Mohamed M. G., Hu H. Y., Madhu M., Samy M. M., Mekhemer I. M. A., Tseng W. L., Chou H. H., Kuo S. W. (2023). Ultrastable two-dimensional
fluorescent conjugated microporous polymers containing pyrene and
fluorene units for metal ion sensing and energy storage. Eur. Polym. J..

[50] M.Ejaz M., Mohamed M. G., Kotp M. G., Elewa A. M., Kuo S. W. (2025). Triphenylamine-linked
triazine (D-A) units based hypercrosslinked porous polymer: Rapid
adsorption and enhanced photodegradation of organic dyes from water. Colloids Surf., A.

[51] Ebrahium S. M., Kao Y. C., El-Bery H. M., Younis O., Mohammed A. A. K., Aly K. I., Kuo S. W., Mohamed M. G. (2025). Rational design
of donor–acceptor (D-A) conjugated microporous polymers containing
thienopyrene and triazine building units for enhanced photocatalytic
hydrogen production. J. Mol. Struct..

[52] Xie P., Han C., Xiang S., Jin S., Ge M., Zhang C., Jiang J. X. (2023). Toward High-Performance
Dibenzo­[g,p]­chrysene-Based
Conjugated Polymer Photocatalysts for Photocatalytic Hydrogen Production
Through Donor-Acceptor-Acceptor Structure Design. Chem. Eng. J..

[53] Ou H., Chen X., Lin L., Fang Y., Wang X. (2018). Biomimetic
Donor–Acceptor Motifs in Conjugated Polymers for Promoting
Exciton Splitting and Charge Separation. Angew.
Chem., Int. Ed..

[54] Shu C., Han C., Yang X., Zhang C., Chen Y., Ren S., Wang F., Huang F., Jiang J. X. (2021). Boosting the photocatalytic
hydrogen evolution activity for D−π–A conjugated
microporous polymers by statistical copolymerization. Adv. Mater..

[55] Samy M. M., Mekhemer I. M. A., Mohamed M. G., Elsayed M. M., Lin K. H., Chen Y. K., Wu T. L., Chou H. H., Kuo S. W. (2022). Conjugated
microporous polymers incorporating Thiazolo [5,4-d] thiazole moieties
for Sunlight-Driven hydrogen production from water. Chem. Eng. J..

[56] Mohamed M. G., Chang S. Y., Ejaz M., Samy M. M., Mousa A. O. K., Kuo S. (2023). Design and synthesis
of bisulfone-linked two-dimensional
conjugated microporous polymers for CO_2_ adsorption and
energy storage. Molecules.

[57] Babejová M., Třísková I., Trnková L., Semrád H., Munzarová M., Heger D., Nachtigallová D., Potáček M. (2024). Synthesis, optical, electrochemical,
and computational study of benzene/thiophene based D−π–A
chromophores. RSC Adv..

[58] Ding H., Chu Y., Xu M., Zhang S., Ye H., Hu Y., Hua J. (2020). Effect of
π-bridge groups based on indeno [1,2-b] thiophene
D–A−π–A sensitizers on the performance
of dye-sensitized solar cells and photocatalytic hydrogen evolution. J. Mater. Chem. C.

[59] Andicsová A. E., Tokárová Z., Kozma E., Balogh R., Vykydalová A., Mróz W., Tokár K. (2023). Thiazolo [5,4-d]
thiazoles with a spirobifluorene moiety as novel D−π–A
type organic hosts: design, synthesis, structure–property relationship
and applications in electroluminescent devices. New J. Chem..

[60] Mohamed M. G., Elsayed M. H., Li C. J., Hassan A. E., Mekhemer I. M. A., Musa A. F., Hussien M. K., Chen L. C., Chen K. H., Chou H. H., Kuo S. W. (2024). Reticular
design and alkyne bridge
engineering in donor−π–acceptor type conjugated
microporous polymers for boosting photocatalytic hydrogen evolution. J. Mater. Chem. A.

[61] Xiao J., Xiao Z., Hu J., Gao X., Asim M., Pan L., Shi C., Zhang X., Zou J. J. (2022). Rational design
of alkynyl-based linear donor– π–acceptor conjugated
polymers with accelerated exciton dissociation for photocatalysis. Macromolecules.

[62] Mohamed M. G., Mekhemer I. M. A., Selim A. F. H., Katsamitros A., Tasis D., Basit A., Chou H. H., Kuo S. W. (2025). Molecular
engineering of donor–acceptor-type conjugated microporous polymers
for dual effective photocatalytic production of hydrogen and hydrogen
peroxide. Mater. Horiz..

[63] Che H., Wang J., Wang P., Ao Y., Chen J., Gao X., Zhu F., Liu B. (2023). Simultaneously
achieving fast intramolecular
charge transfer and mass transport in holey D−π–A
organic conjugated polymers for highly efficient photocatalytic pollutant
degradation. JACS Au.

[64] Hou Y., Liu F., Zhang B., Tong M. (2022). Thiadiazole-based covalent organic
frameworks with a donor–acceptor structure: modulating intermolecular
charge transfer for efficient photocatalytic degradation of typical
emerging contaminants. Environ. Sci. Technol..

[65] Basit A., Kao Y. C., El-Ossaily Y. A., Kuo S. W., Mohamed M. G. (2024). Rational
engineering and synthesis of pyrene and thiazolo [5,4-d] thiazole-functionalized
conjugated microporous polymers for efficient supercapacitor energy
storage. J. Mater. Chem. A.

[66] Wang H., Almatrafi E., Wang Z., Yang Y., Xiong T., Yu H., Qin H., Yang H., He Y., Zhou C., Zeng G. (2022). Self-assembly
hybridization of COFs and g-C_3_N_4_: decipher the
charge transfer channel for enhanced photocatalytic
activity. J. Colloid Interface Sci..

[67] Mekhemer I. M. A., Elewa A. M., Elsenety M. M., Samy M. M., Mohamed M. G., Musa A. F., Huang T. F., Wei T. C., Kuo S. W., Chen B. H. (2024). Self-condensation
for enhancing the hydrophilicity
of covalent organic polymers and photocatalytic hydrogen generation
with unprecedented apparent quantum yield up to 500 nm. Chem. Eng. J..

[68] Liang Y. C., Chen B. Y. (2023). Enhanced photocatalytic
activity of Ag_2_S
particle decorated S-doped WO_3_ nanorods synthesized through
two-stage vaporous vulcanization processes. CrystEngcomm.

[69] Salahshoori I., Wang Q., Nobre M. A., Mohammadi A. H., Dawi E. A., Khonakdar H. A. (2024). Molecular
simulation-based insights
into dye pollutant adsorption: a perspective review. Adv. Colloid Interface Sci..

[70] Kao Y. C., Yeh K. T., Mohamed M. G., Karim H., Su W. H., Kuo S. W. (2025). Structural modulation via mesoporous
silica templating
in covalent organic frameworks: Converting functional aspects for
adsorption behavior. Sep. Pur. Technol..

[71] Du X. C., Zhu J. H., Quan Z. I., Wang X. C. (2021). Adsorption of rhodamine
B by organic porous materials rich in nitrogen, oxygen, and sulfur
heteroatoms. New J. Chem..

[72] Pan X., Qin X., Zhang Q., Ge Y., Ke H., Cheng G. (2020). N-and S-rich
covalent organic framework for highly efficient removal of indigo
carmine and reversible iodine capture. Microporous
Mesoporous Mater..

[73] Hazra A., Samanta S. K. (2025). Fabricating Tetraphenylethylene-Based Ionic Porous
Organic Polymers for Efficient Sequestration of Toxic Iodine and Oxoanions
in Multiple Media. ACS Appl. Mater. Interfaces.

[74] Khan T. A., Dahiya S., Ali I. (2012). Use of kaolinite
as adsorbent: Equilibrium,
dynamics and thermodynamic studies on the adsorption of Rhodamine
B from aqueous solution. Appl. Clay Sci..

[75] Sharma K., Sudhaik A., Raizada P., Thakur P., Pham X. M., Van Le Q., Nguyen V. H., Ahamad T., Thakur S., Singh P. (2023). Constructing α-Fe_2_O_3_/g-C_3_N_4_/SiO_2_ S-scheme-based heterostructure for photo-Fenton
like degradation of rhodamine B dye in aqueous solution. Environ. Sci. Pollut. Res..

