# Supplementary material for: Synergistic Engineering of Pyrene–Thiazolothiazole-Based Donor−π–Acceptor Conjugated Microporous Polymers with Heteroatom Embedding for Efficient Visible-Light Photocatalyst for Organic Dye Degradation
Source: ACS Polym Au. 2025 Sep 11;5(5):633–44. doi: 10.1021/acspolymersau.5c00083 (PMC12511974; doi:10.1021/acspolymersau.5c00083)
Supplement: Supplementary file 1 [file lg5c00083_si_001.pdf]

Supporting Information

Synergistic Engineering of Pyrene–Thiazolothiazole–Based Donor–π–Acceptor Conjugated Microporous Polymers with Heteroatom Embedding for Efficient Visible-Light Photocatalyst for Organic Dye Degradation

Yang-Chin Kao[a#], Mohamed Gamal Mohamed[a,b#,*], Ying-Hong Chen[a], Mohsin Ejaz[a], and Shiao-Wei Kuo[a,c*]

[a]Department of Materials and Optoelectronic Science, Center for Functional Polymers and Supramolecular Materials, National Sun Yat-Sen University, Kaohsiung 804, Taiwan.

[b]Department of Chemistry, Faculty of Science, Assiut University, Assiut 71515, Egypt.

[c]Department of Medicinal and Applied Chemistry, Kaohsiung Medical University, Kaohsiung 807, Taiwan.

<u>**Corresponding authors:**</u>

E-mail: mgamal.eldin12@yahoo.com, mgaml.eldin12@aun.edu.eg (M. G. Mohamed), and kuosw@faculty.nsysu.edu.tw (S. W. Kuo).

[#]These authors contributed equally.

Characterization

FTIR spectra were collected on a Bruker Tensor 27 FTIR spectrophotometer with a resolution of 4 cm^{-1} by using the KBr disk method. ^{13}C nuclear magnetic resonance (NMR) spectra were examined by using an INOVA 500 instrument with DMSO-d_6 and $CDCl_3$-d as the solvents and TMS as the external standard. Chemical shifts are reported in parts per million (ppm). The thermal stabilities of the samples were performed by using a TG Q-50 thermogravimetric analyzer under a N_2 atmosphere; the sample (ca. 5 mg) was put in a Pt cell with a heating rate of 20 °C min^{-1} from 100 to 800 °C under a N_2 flow rate of 60 mL min^{-1}. Solid-state ^{13}C NMR was measured by JEOL JNM-LA300 spectrometer and a standard CPMAS probe at 75.577 MHz. The morphologies of the polymer network samples were examined by Field emission scanning electron microscopy (FE-SEM; JEOL JSM7610F). Surface area and porosity measurements of samples weighing approximately 40-60 mg were conducted using the BEL MasterTM/BEL simTM (version 3.0.0) apparatus. Nitrogen (N_2) adsorption and desorption isotherms were generated by gradually exposing the samples to ultrahigh-purity N_2 gas, reaching pressures of up to about 1 atmosphere, while maintaining a temperature of 77 K in a liquid nitrogen bath. Before these measurements, the samples underwent a degassing process at 150 °C for 8 h. The instrument's software was utilized to calculate surface parameters using the BET adsorption models. Furthermore, the pore size of the prepared samples was determined using the nonlocal density functional theory (NLDFT). Optical properties were characterized by photoluminescence (PL) spectroscopy on a Hitachi F-7000 fluorescence spectrophotometer. UV-Vis diffuse reflectance spectra (DRS) were recorded on a JASCO V-770 UV-Vis/NIR spectrophotometer to estimate the optical band gaps of the materials via Tauc plot analysis. The highest occupied molecular orbital (HOMO) energy levels were evaluated using ultraviolet photoelectron

spectroscopy (UPS) on a PHI VersaProbe 4 system. Surface chemical compositions and elemental valence states were analyzed by X-ray photoelectron spectroscopy (XPS) using a ULVAC-PHI (Quantes) instrument. The photodegradation products of Rhodamine B were identified by liquid chromatography–mass spectrometry (LC-MS) using a Shimadzu MALDI-7090 TOF/TOF mass spectrometer. Lastly, the formation of reactive radical species under light irradiation was verified by electron paramagnetic resonance (EPR) spectroscopy using a EMXplus-10/12/P/L SYSTEM.

Adsorption and degradation kinetics

Adsorption Kinetics: The removal efficiency (%) was calculated using the following equation:

$$[(C_0 - C_t)/C_0] \times 100\%$$

Where C_0 (mg/L) is the initial concentration, and C_t (mg/L) is the remaining concentration of the pollutant. The amount of RhB adsorbed at different temperatures was determined using the Van't Hoff equation:

$$\ln K_C = -\frac{\Delta H}{RT} + \frac{\Delta S}{R}$$

Where K_c is the equilibrium constant, which can be expressed as q_e/C_e, R is the universal gas constant [8.314 J/(mol · K)], and T is the absolute temperature in Kelvin (K). By applying this equation, the enthalpy change ($\Delta H°$) and entropy change ($\Delta S°$) of the adsorption process can be determined.

The degradation kinetics were analyzed using a pseudo-first-order model, which is expressed by the equation:

$$ln(C_0' \,/\, C_t) = kt$$

Here, C_0' (mg/L) represents the pollutant concentration at the onset of light irradiation in 0 min,

C_t (mg/L) is the concentration remaining after a reaction time t, and k (min^{-1}) is the apparent rate constant. By plotting ln (C_0'/ C_t) against t, a linear relationship is obtained, and the slope of this line corresponds to k. This approach allows straightforward comparison of photocatalytic activities under different experimental conditions.

Photocatalytic experiment

To investigate the photocatalytic performance of the catalyst, RhB degradation under visible light irradiation was examined. In a typical experiment, Pyr-Ph-TzTz CMP or Pyr-Th-TzTz CMP (10 mg) was dispersed in an aqueous RhB solution (20 mL, 30 ppm) and stirred in the dark for 30 min to achieve adsorption-desorption equilibrium. Subsequently, the suspension was irradiated with a 300 W xenon lamp equipped with a filter ($\lambda > 400$ nm).

In ultraviolet photoelectron spectroscopy (UPS) and Tauc plot analysis, the obtained energy values are expressed in electronvolts (eV) with respect to the vacuum level. On the other hand, the redox potentials such as $O_2/{\bullet}O_2^-$ and $H_2O/{\bullet}OH^-$ are reported in volts (V) relative to a reference electrode (e.g., NHE). The conversion between these two scales is performed by referencing the vacuum level to the electrochemical scale. Conventionally, the potential of the normal hydrogen electrode (NHE) is taken as −4.5 eV vs. vacuum. Thus, the energy levels in eV can be converted to electrochemical potentials in V by the following relation:

$$E \text{ (vs. NHE, V)} = -[E \text{ (vs. vacuum, eV)} + 4.5]$$

This allows direct comparison between the electronic band positions (in eV) and the redox potentials (in V).

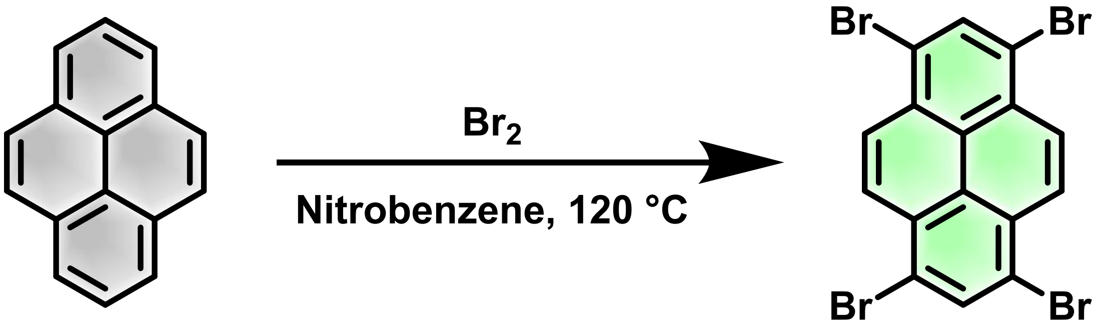

Scheme S1. Synthesis of 1,3,6,8-tetrabromopyrene (Pyr-4Br).

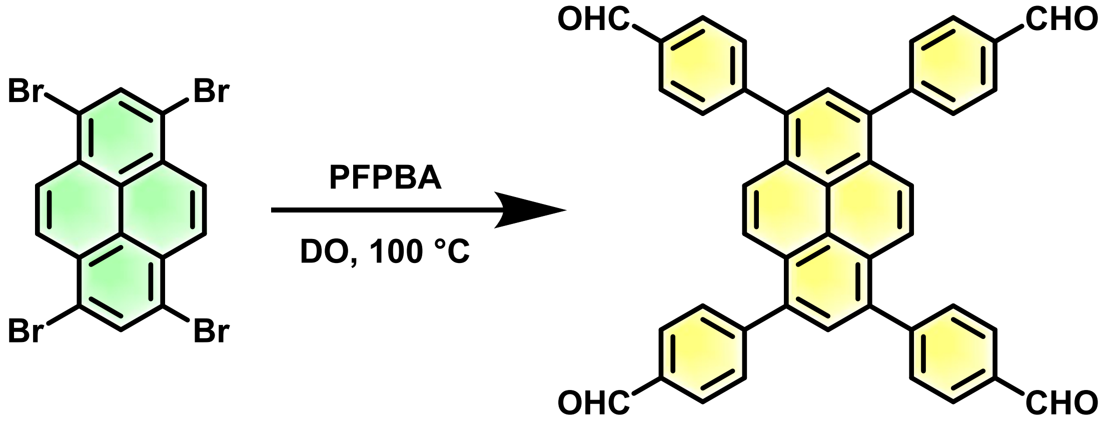

Scheme S2. Synthesis of 4,4',4'',4'''-(pyrene-1,3,6,8-tetrayl)tetrabenzaldehyde (Pyr-4Ph-CHO).

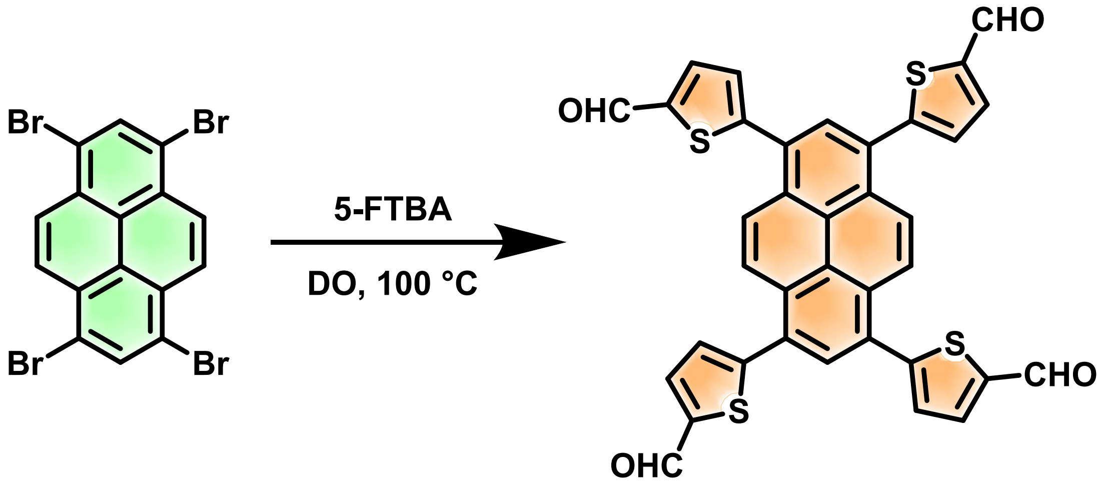

Scheme S3. Synthesis of 5,5',5'',5'''-(pyrene-1,3,6,8-tetrayl)tetrakis(thiophene-2-carbaldehyde) (Pyr-4Th-CHO).

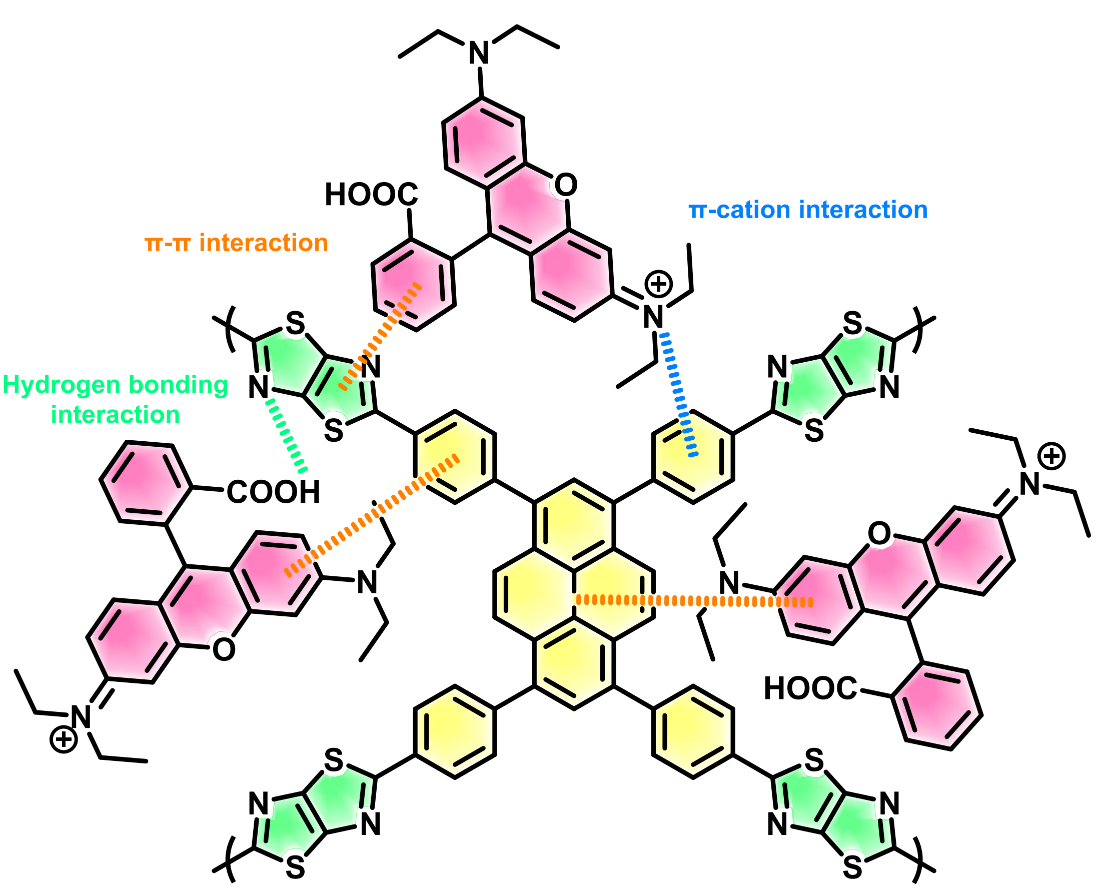

Scheme S4. Possible RhB adsorption mechanisms of Pyr-Ph-TzTz CMP.

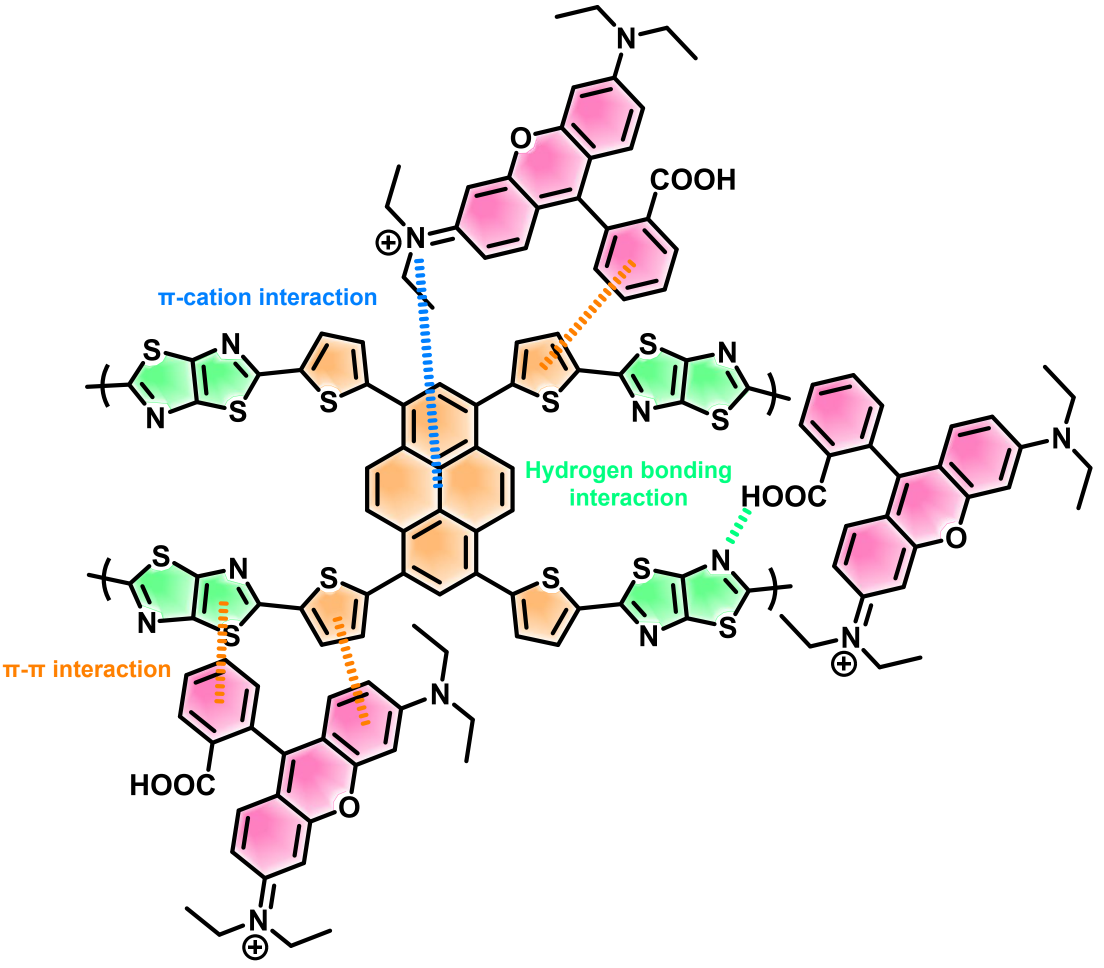

Scheme S5. Possible RhB adsorption mechanisms of Pyr-Th-TzTz CMP.

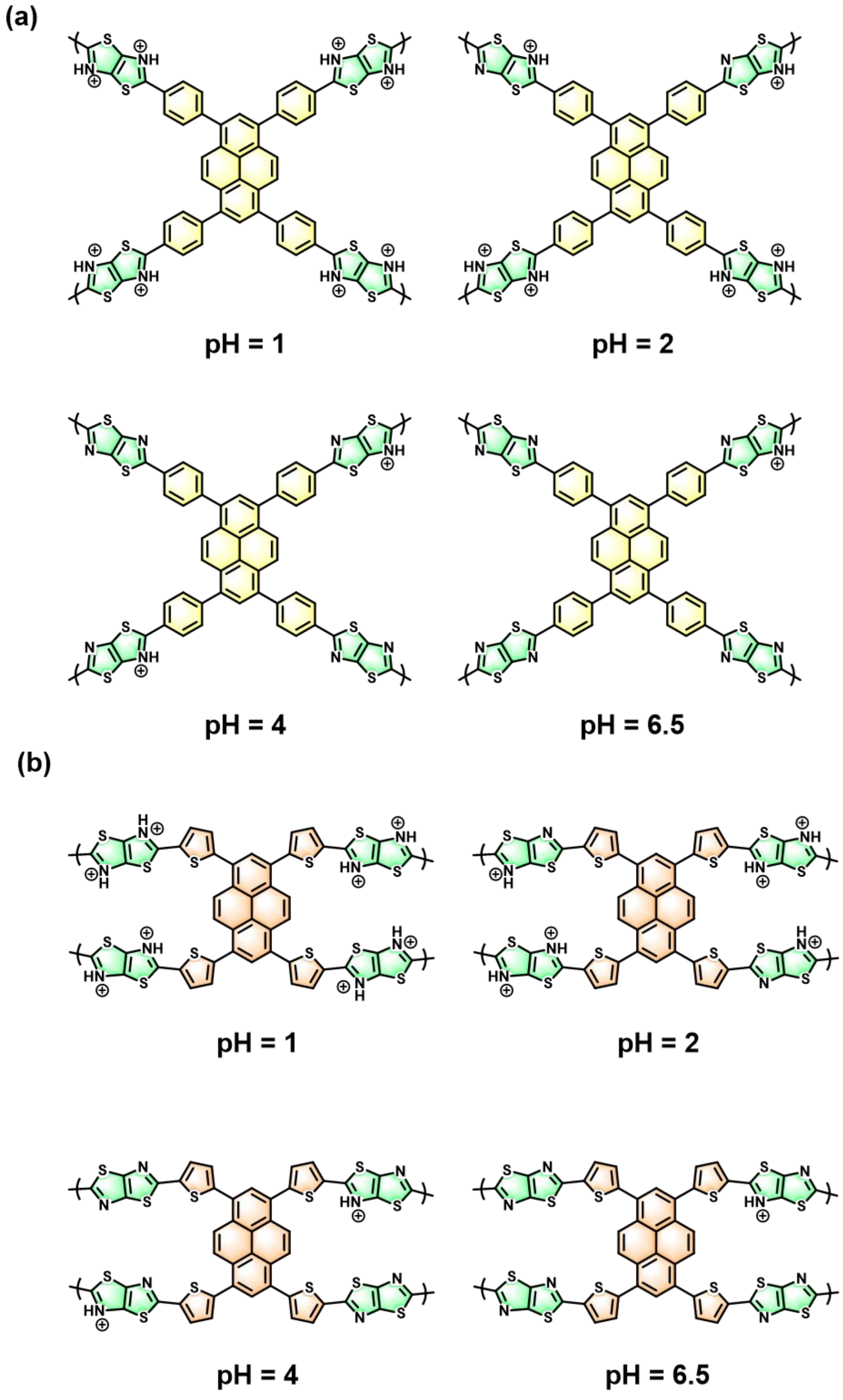

Scheme S6. Possible structure of (a) Pyr-Ph-TzTz and (b) Pyr-Th-TzTz CMPs in different pH values.

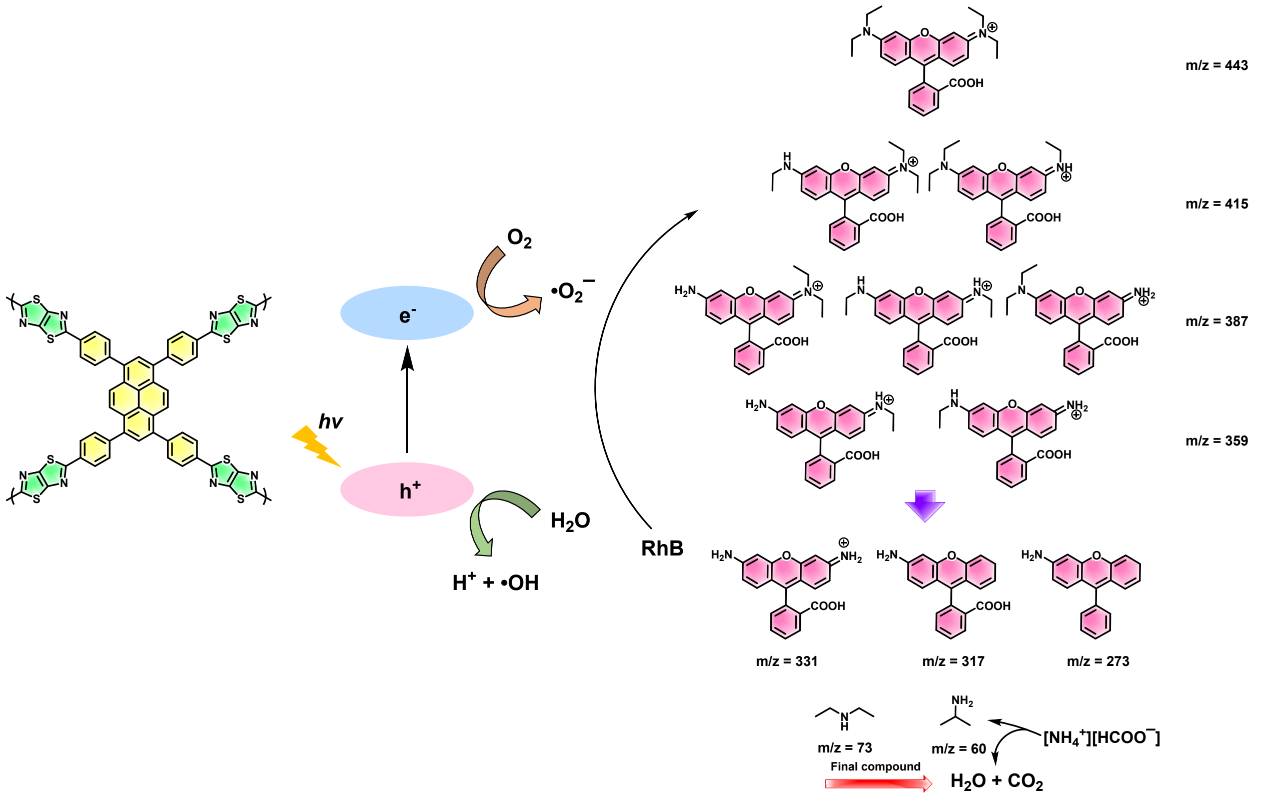

Scheme S7. Possible photocatalytic degradation pathways of RhB with Pyr-Ph-TzTz CMP.

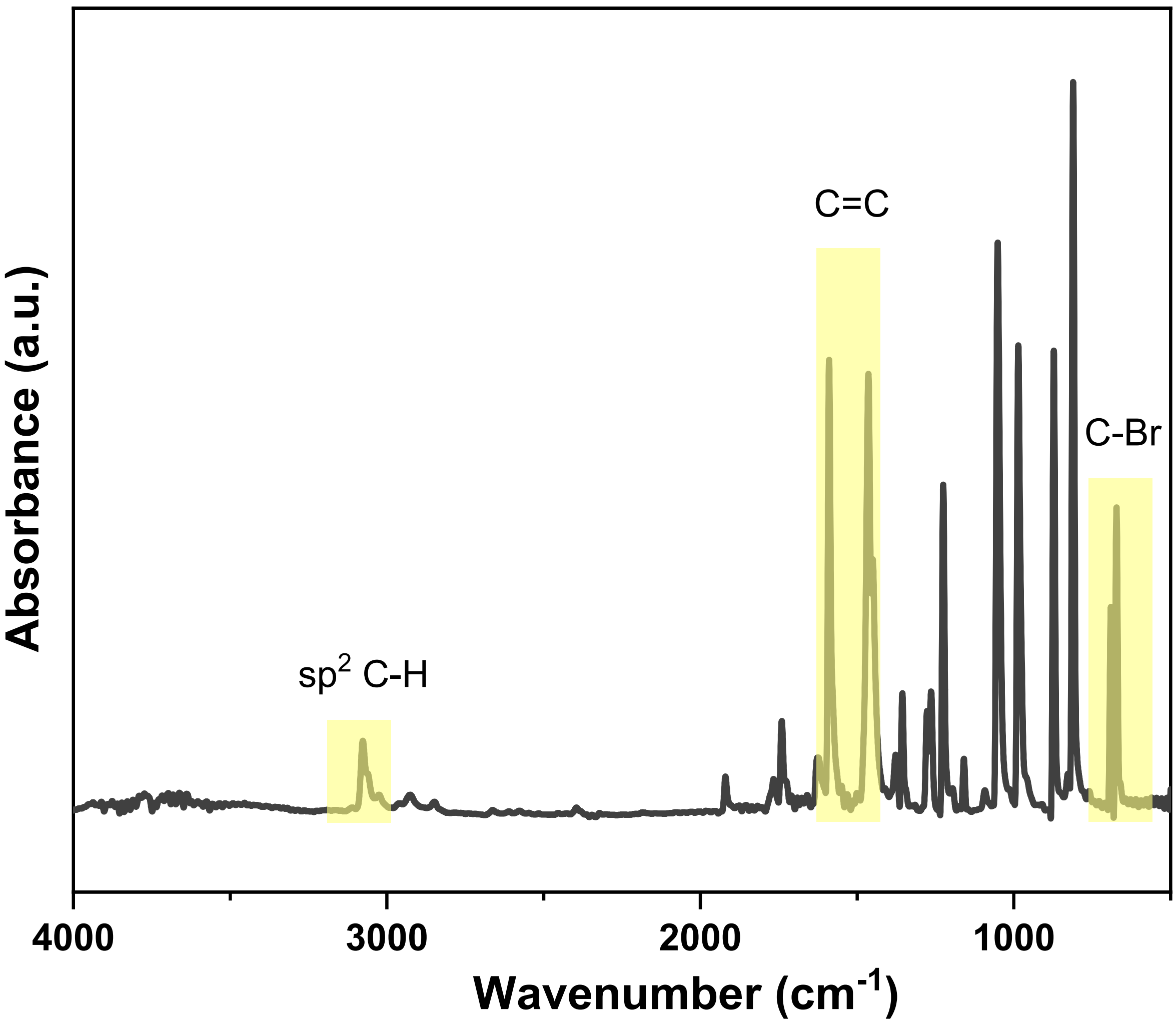

Figure S1. FT-IR spectra of Pyr-4Br.

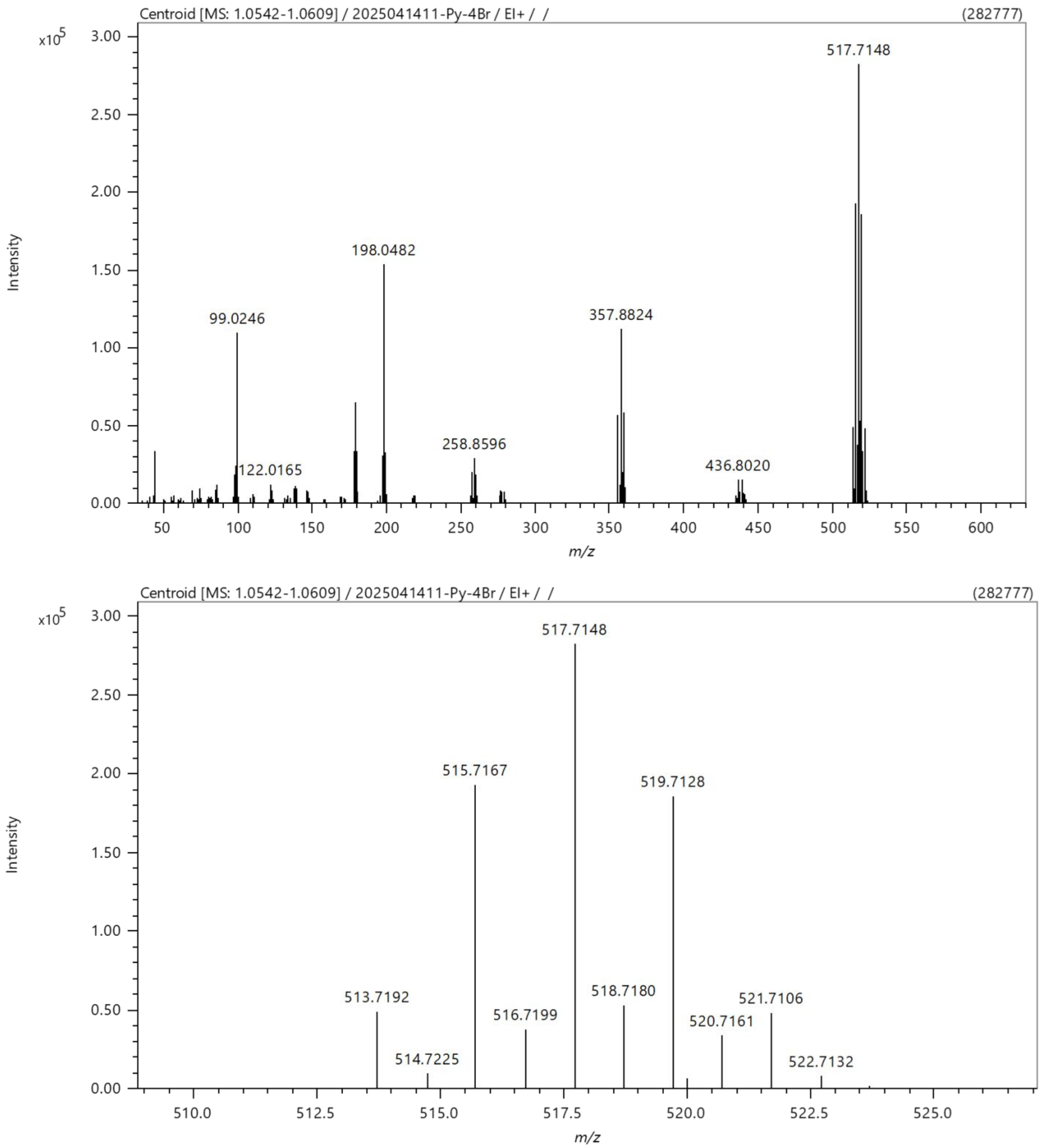

Figure S2. HREI-MS spectra of Pyr-4Br.

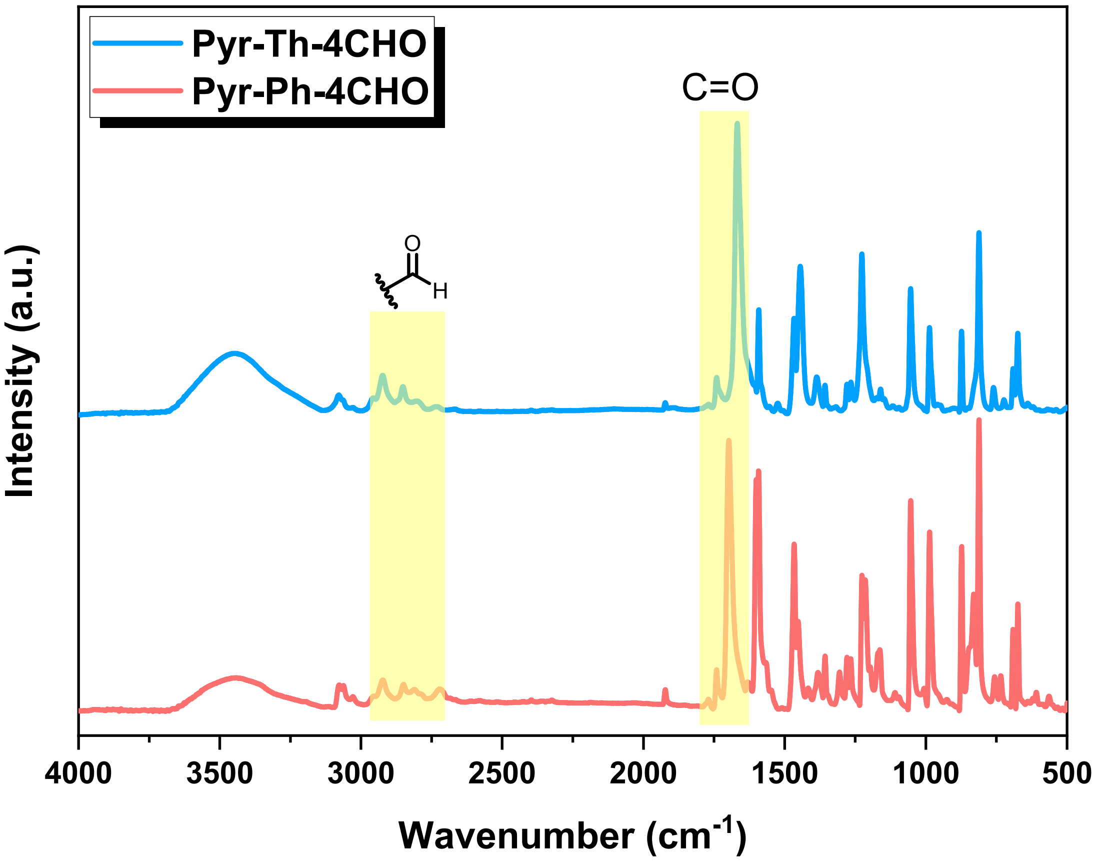

Figure S3. FT-IR spectra of Pyr-Ph-4CHO and Pyr-Th-4CHO.

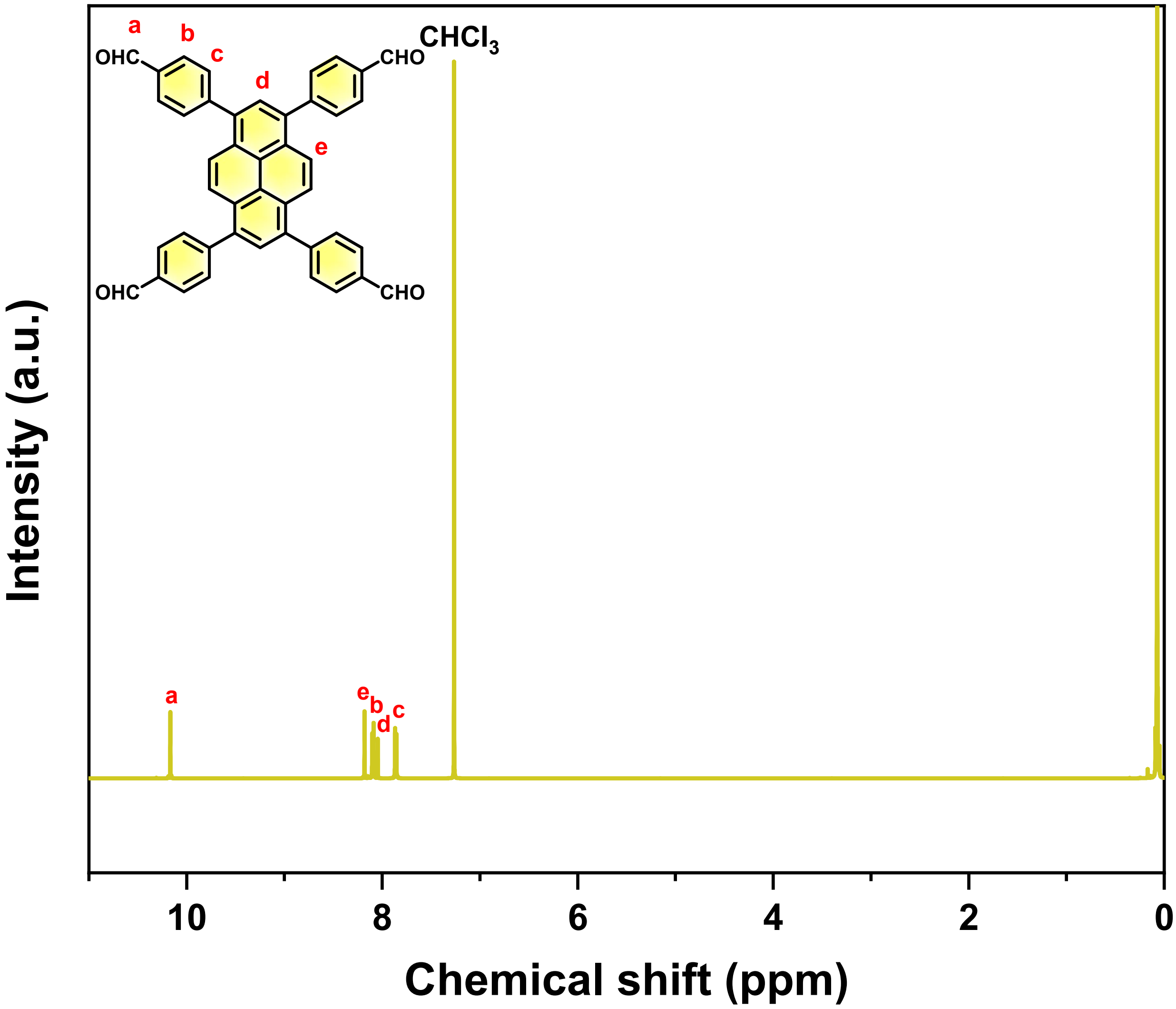

Figure S4. ^{1}H-NMR spectrum of Pyr-Ph-4CHO.

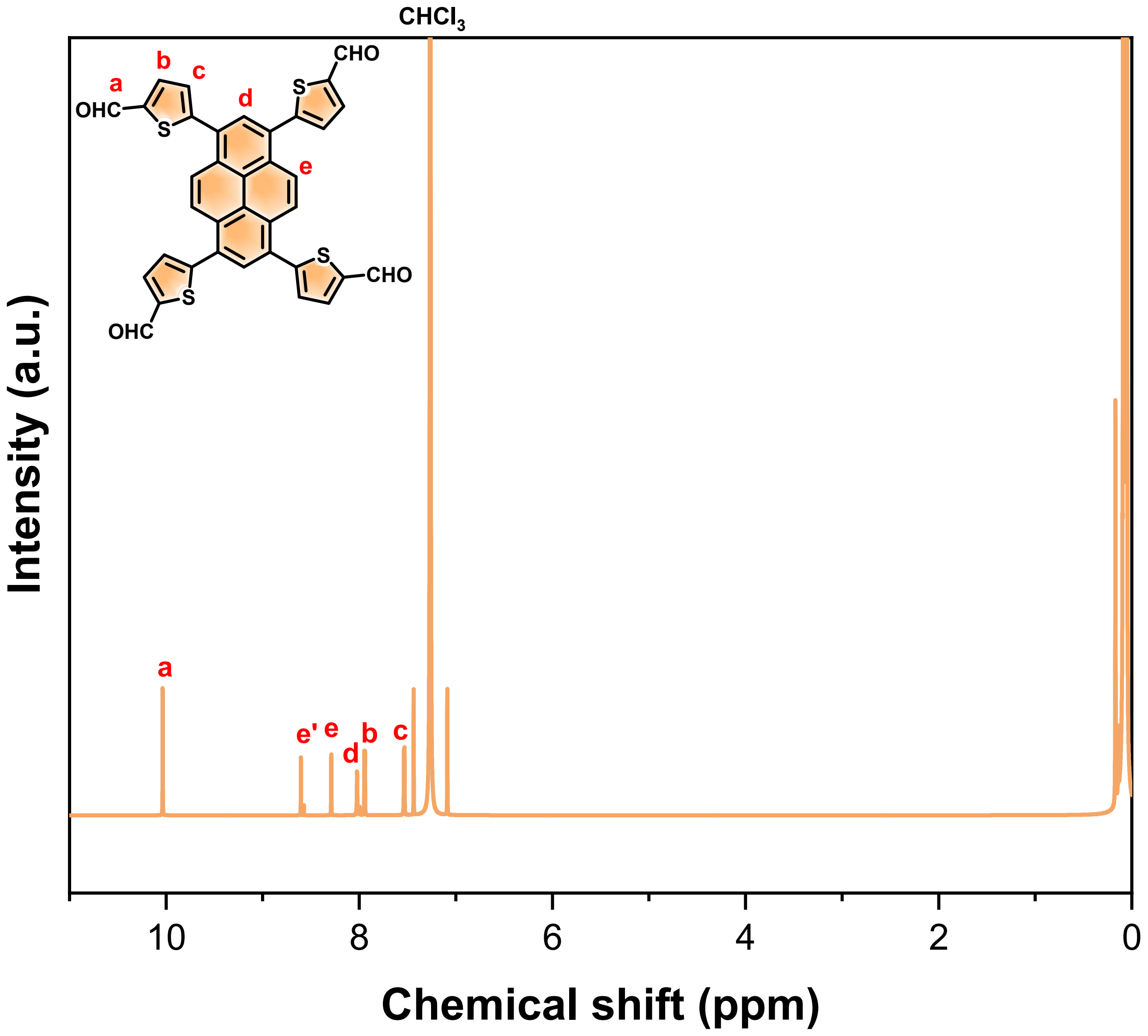

Figure S5. ^{1}H-NMR spectrum of Pyr-Th-4CHO.

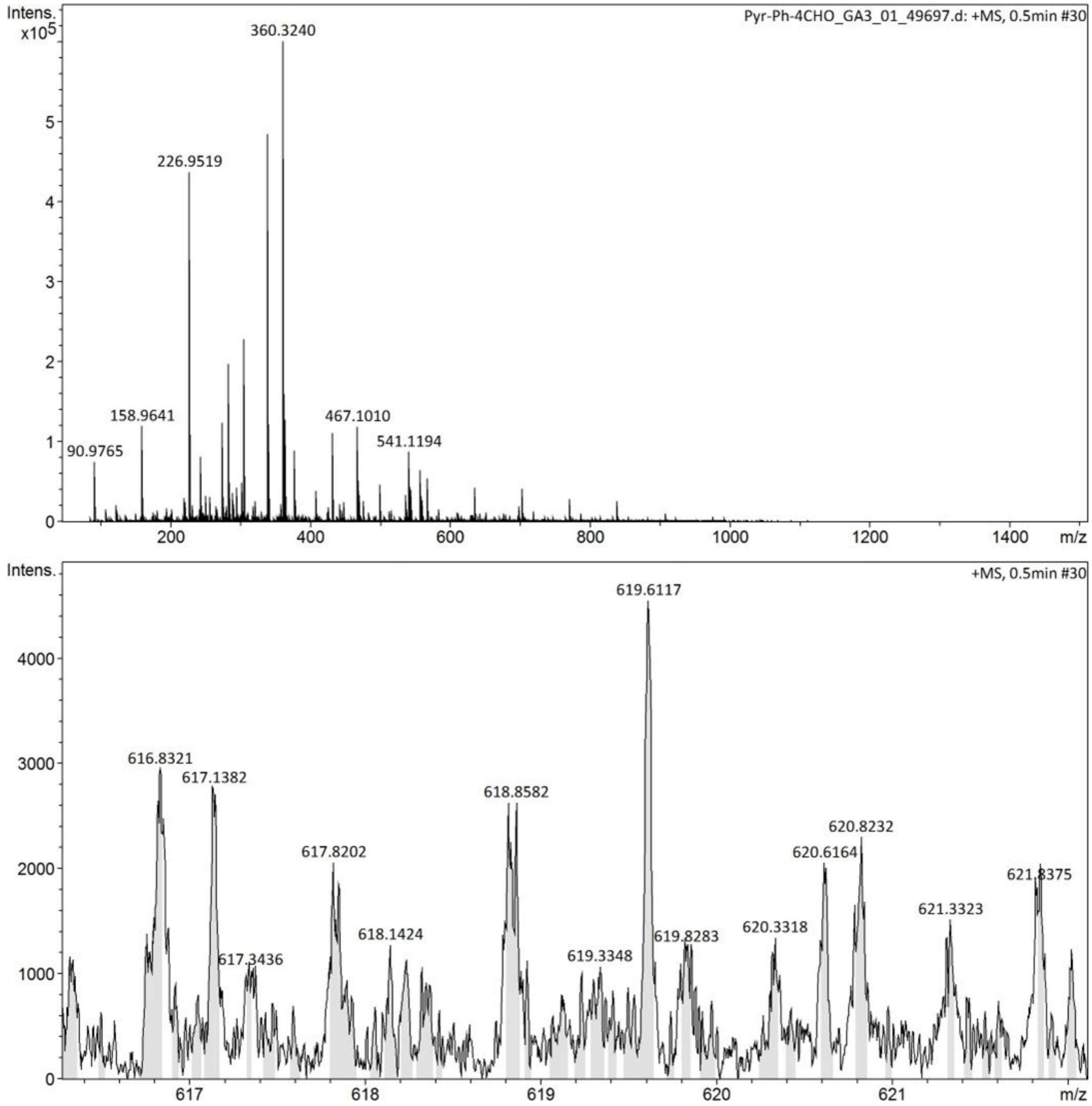

Figure S6. ESI-MS (positive mode) profiles of Pyr-Ph-4CHO.

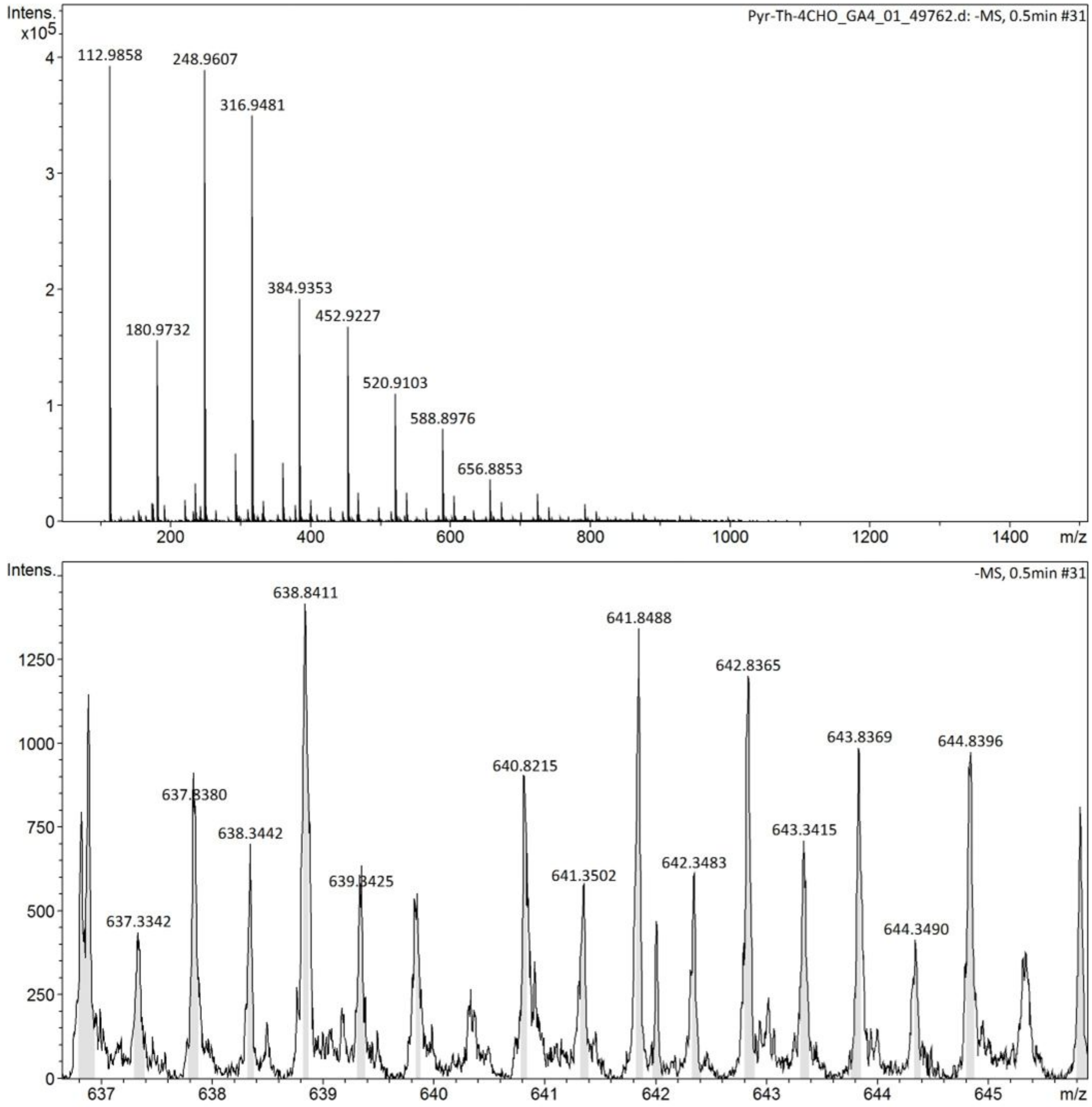

Figure S7. ESI-MS (negative mode) profiles of Pyr-Th-4CHO.

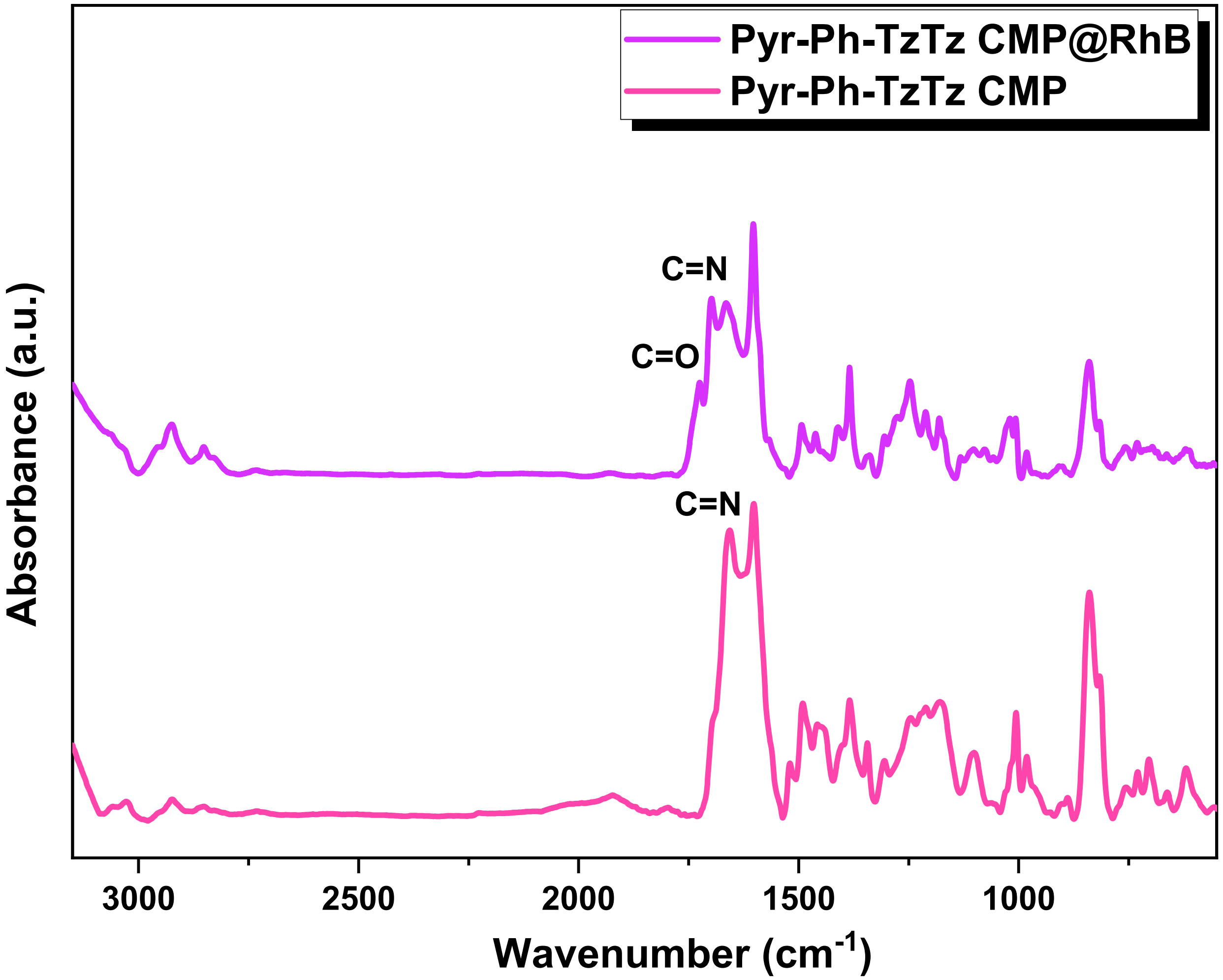

Figure S8. FT-IR spectra of Pyr-Ph-TzTz CMP before and after RhB adsorption.

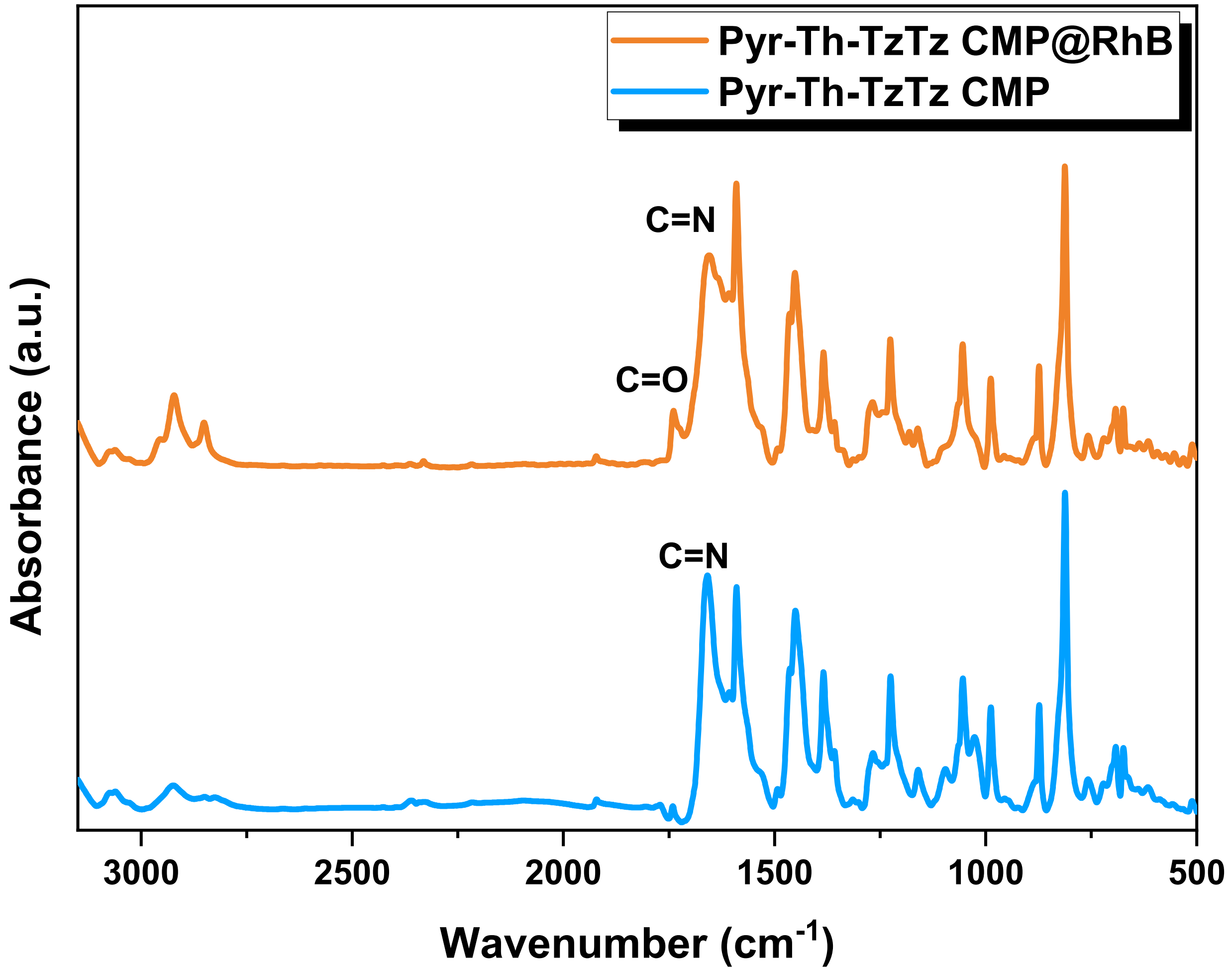

Figure S9. FT-IR spectra of Pyr-Th-TzTz CMP after RhB adsorption.

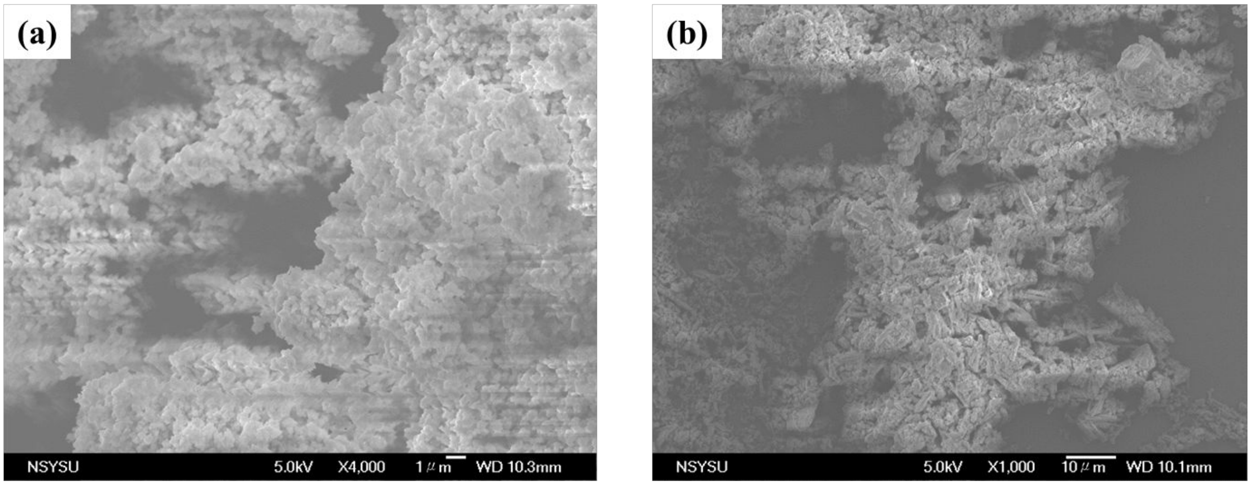

Figure S10. SEM image of (a) Pyr-Ph-TzTz and (b) Pyr-Th-TzTz CMPs after RhB adsorption.

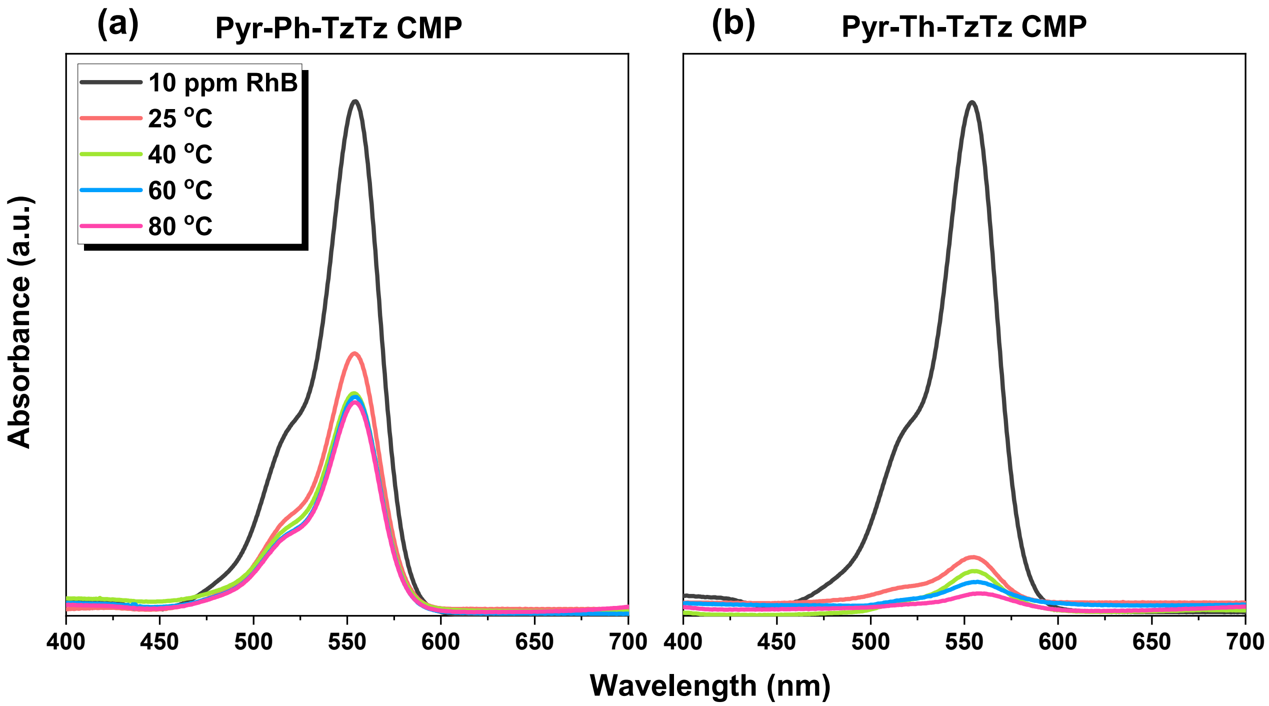

Figure S11. UV-Vis spectra of (a) Pyr-Ph-TzTz CMP and (b) Pyr-Th-TzTz CMP at different temperatures with 10 ppm RhB solution.

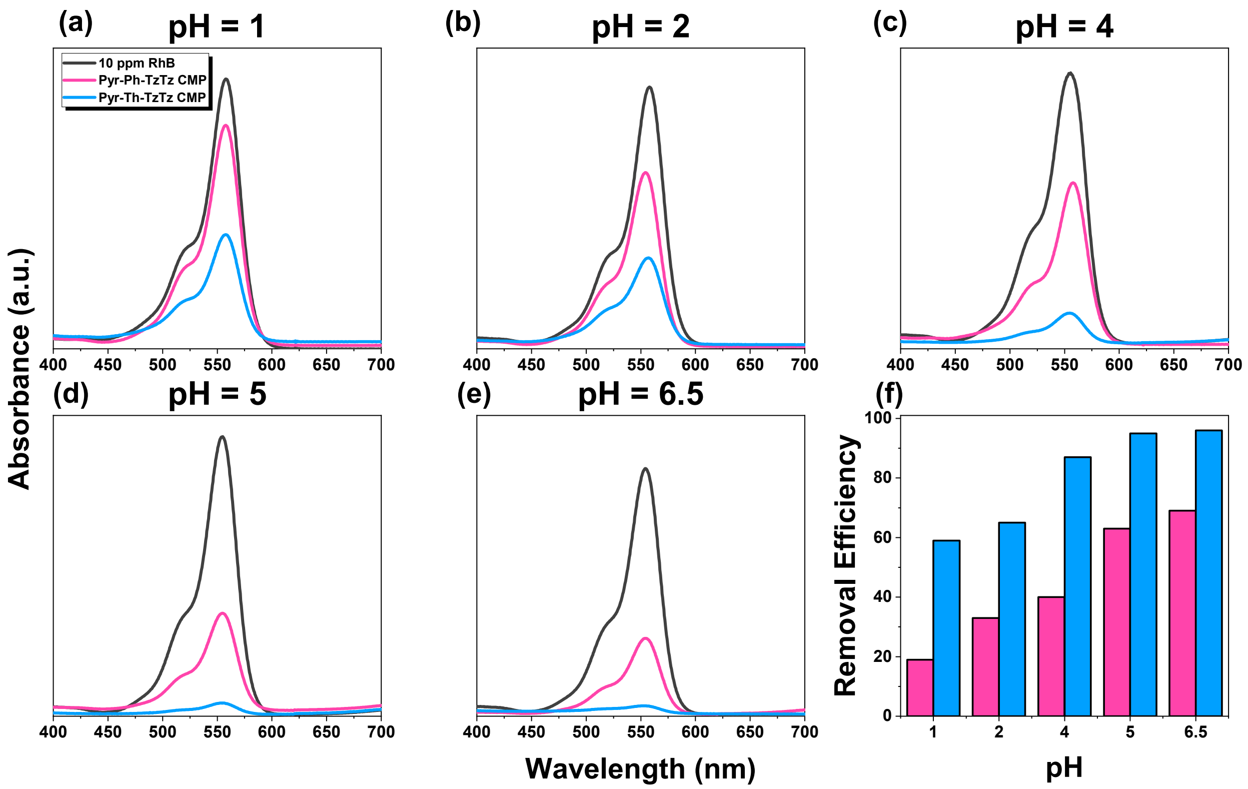

Figure S12. UV-Vis spectra of thiazolothiazole-linked CMPs in pH = (a) 1, (b) 2, (c) 4, (d) 5, and (e) 6.5 with 10 ppm RhB solution and (f) the summarized removal efficiency of Pyr-Ph-TzTz CMP and Pyr-Ph-TzTz CMP in different pH values.

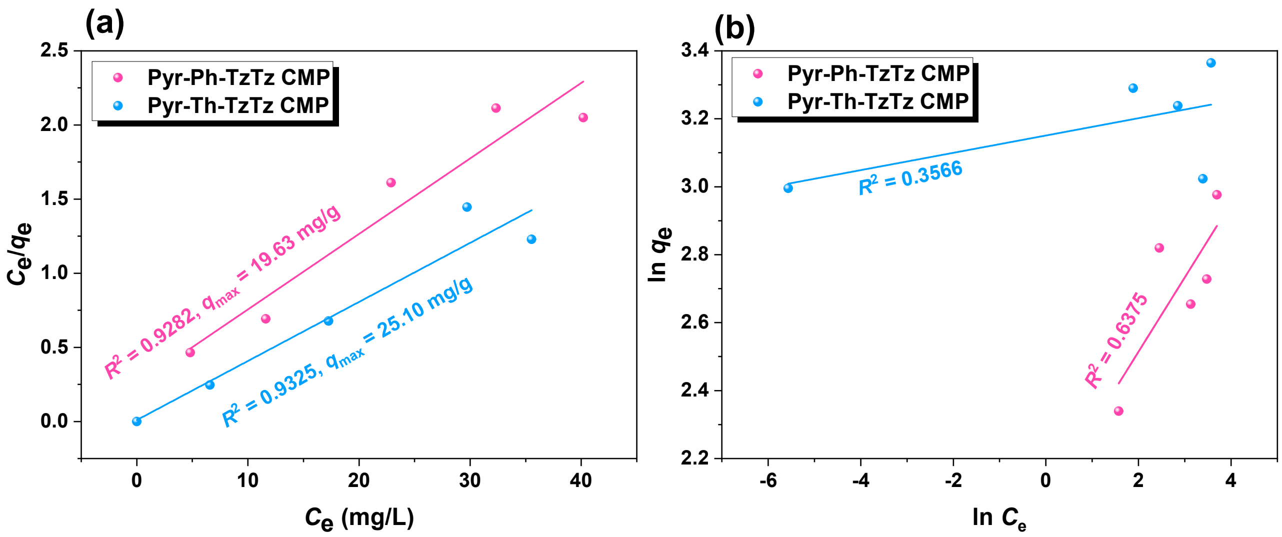

Figure S13. (a) Langmuir fitting and (b) Freundlich fitting of Pyr-Ph-TzTz CMP and Pyr-Th-TzTz CMP.

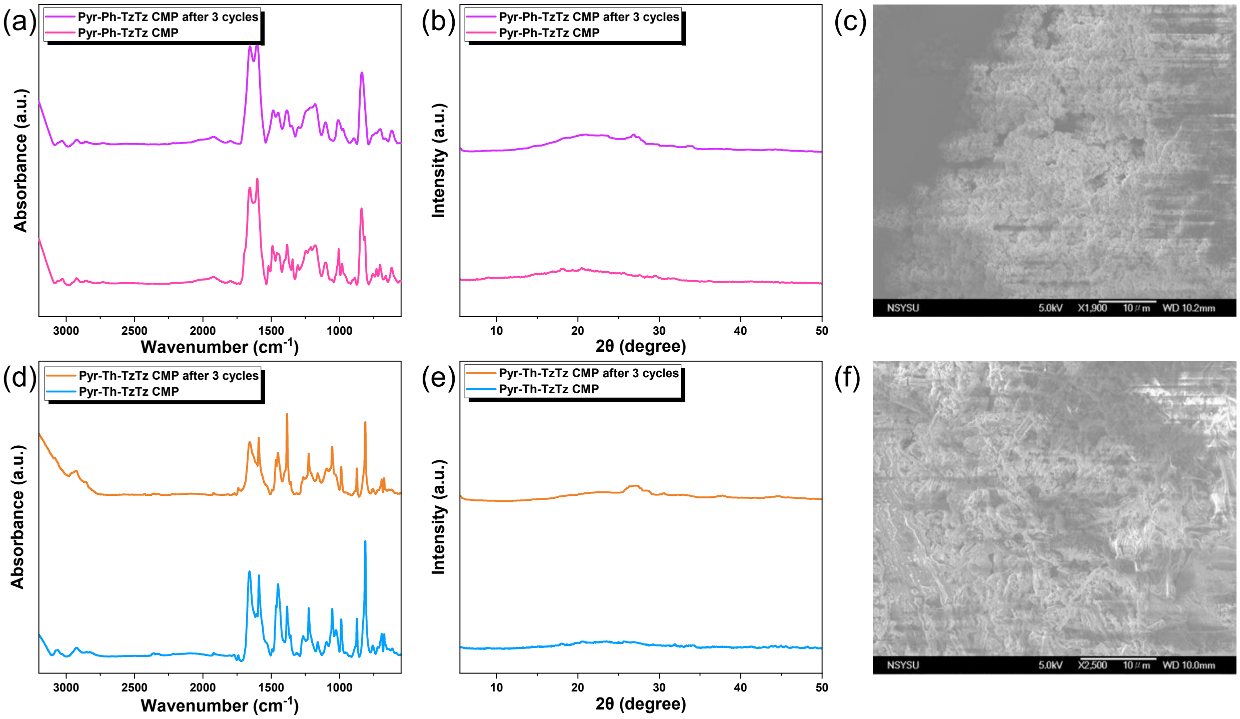

Figure S14. FTIR spectra of (a) Pyr-Ph-TzTz and (d) Pyr-Th-TzTz CMPs. XRD profiles of (b) Pyr-Ph-TzTz and (e) Pyr-Th-TzTz CMPs. SEM images of (c) Pyr-Ph-TzTz and (f) Pyr-Th-TzTz CMP after 3 cycles.

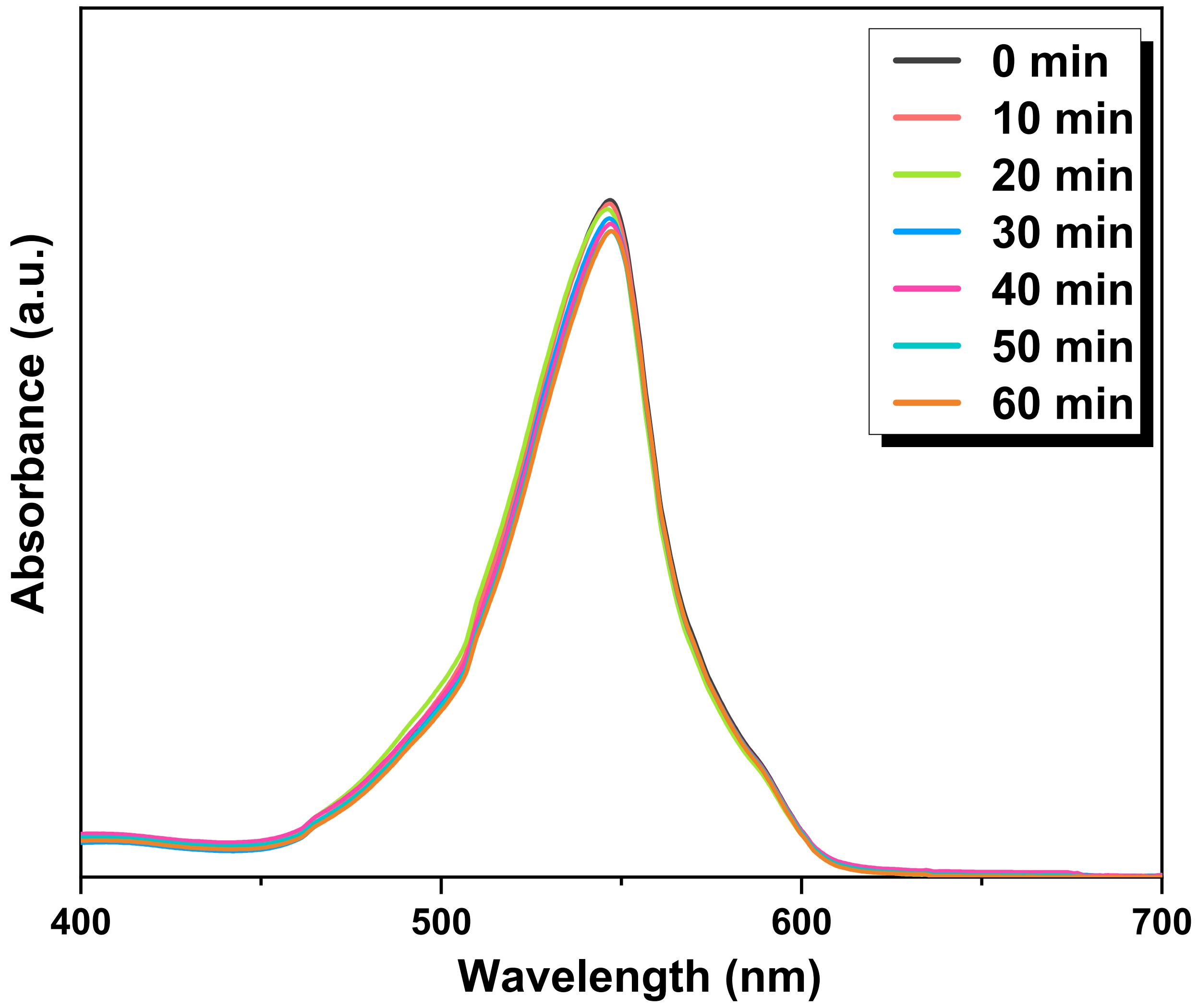

Figure S15. UV spectra of Rhodamine B dye under visible light irradiation for different times.

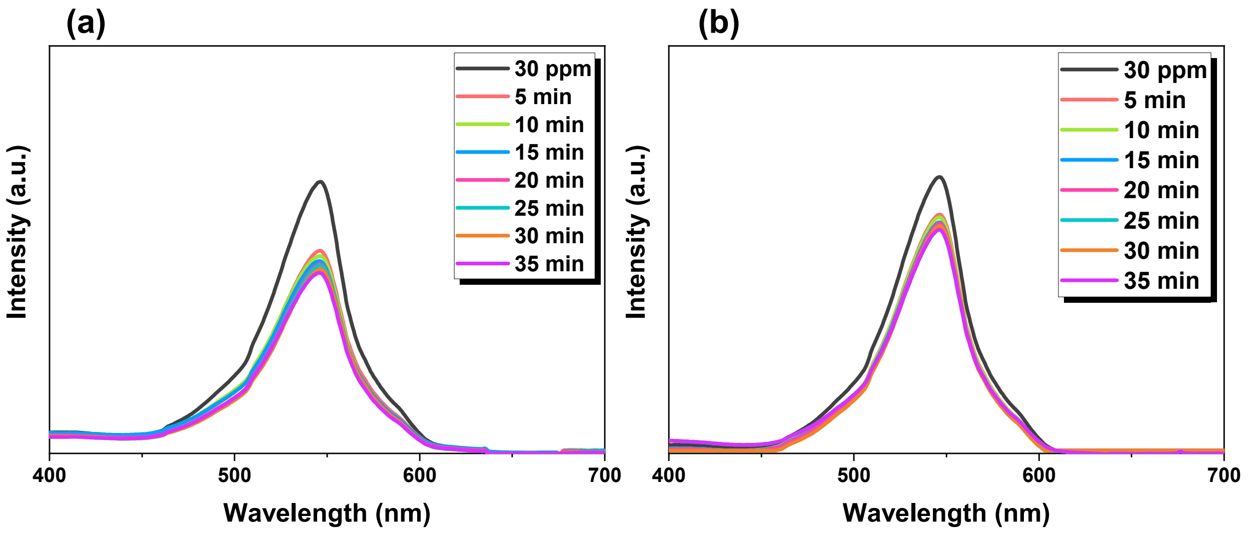

Figure S16. UV spectra of (a) Pyr-Ph-TzTz CMP and (b) Pyr-Th-TzTz CMP adsorption in a dark room for 35 minutes.

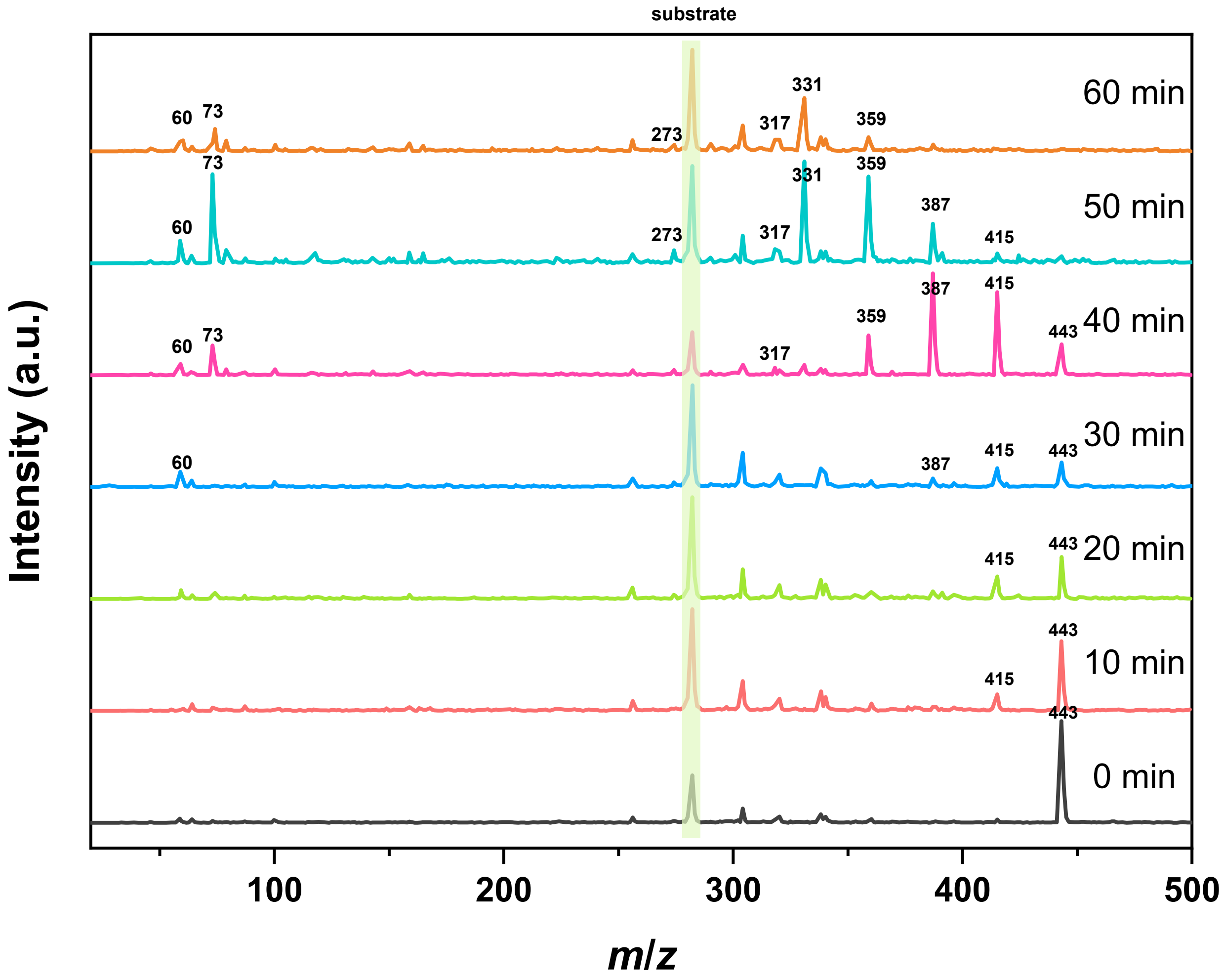

Figure S17. LC-MS analysis of photodegradation of RhB over Pyr-Ph-TzTz CMP under light irradiation. (Peak at m/z = 282 is from the background of the substrate).

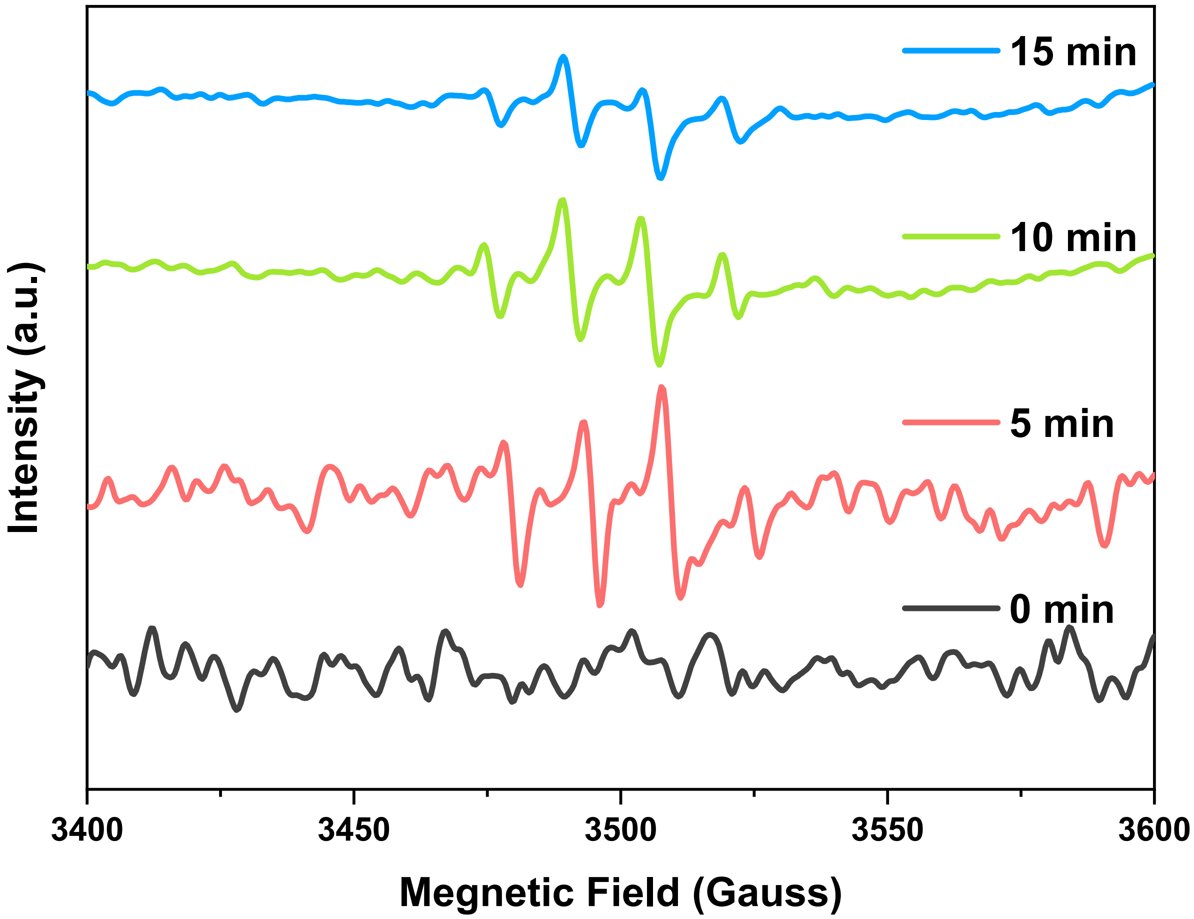

Figure S18. EPR signals of •OH by DMPO-OH with Pyr-Ph-TzTz CMP.

Table S1. The atomic weight percentages of carbon (C), nitrogen (N), and sulfur (S) atoms in the Pyr-Ph-TzTz CMP and Pyr-Th-TzTz CMP.

Sample	C (%)	N (%)	S (%)
**Pyr-Ph-TzTz CMP**	**83.89**	**9.34**	**6.76**
**Pyr-Th-TzTz CMP**	**81.44**	**9.95**	**8.61**

Table S2. Thermodynamic parameters of RhB dye adsorption onto Pyr-Ph-TzTz CMP and Pyr-Th-TzTz CMP.

<table>
<tr><th colspan="2">Sample</th><th>Pyr-Ph-TzTz CMP</th><th>Pyr-Th-TzTz CMP</th></tr>
<tr><td colspan="2">ΔH (kJ/mol)</td><td>25.153</td><td>254.210</td></tr>
<tr><td colspan="2">ΔS (kJ/mol K)</td><td>0.112</td><td>1.104</td></tr>
<tr><td rowspan="4">ΔG (kJ/mol)</td><td>298 K</td><td>-8.223</td><td>-74.781</td></tr>
<tr><td>313 K</td><td>-9.903</td><td>-91.341</td></tr>
<tr><td>333 K</td><td>-12.143</td><td>-113.421</td></tr>
<tr><td>353 K</td><td>-14.383</td><td>-135.501</td></tr>
</table>

Table S3. The maximum adsorption capacities of Pyr-Ph-TzTz CMP and Pyr-Th-TzTz CMP were summarized at different RhB concentrations.

	C_e (mg/L)	Q_e (mg/g)
**Pyr-Ph-TzTz CMP**	4.14753	11.70493
	12.46335	12.83084
	23.28323	13.43354
	32.26219	15.07329
	43.58458	15.47562
**Pyr-Th-TzTz CMP**	**C_e (mg/L)**	**Q_e (mg/g)**
	$3.30314E^{-4}$	19.99934
	7.06522	20.70868
	17.55136	22.92103
	29.64566	24.89729
	38.53948	24.86955
